# Resolving ion-coupled electron transfer in bipolar organic electrodes in aqueous electrolytes by single-particle electrochemistry

**DOI:** 10.1039/d6sc04619c

**Published:** 2026-09-25

**Authors:** George A. Akpowu, John R. Castilon, Pelumi Adanigbo, Andrea B. Brothers, Thida Samay, Victor Wang, Chao Luo, Yun Yu

**Affiliations:** a Department of Chemistry and Biochemistry, George Mason University Fairfax Virginia 22030 USA yyu26@gmu.edu; b Department of Chemical, Environmental and Materials Engineering, University of Miami Coral Gables Florida 33146 USA

## Abstract

Bipolar organic electrode materials, integrating both n-type and p-type redox centers within a single molecular framework, offer compelling opportunities for high-capacity aqueous energy storage. Yet the ion-coupled electron transfer mechanisms that govern each center in aqueous environments are still not fully understood. Here, we employ scanning electrochemical cell microscopy (SECCM) to interrogate bipolar redox processes in a perylene diimide–triphenylamine (PDI–TPA) organic electrode at the single-particle level in aqueous media. Electrochemical mapping directly reveals sub-particle heterogeneity governed by particle thickness, establishing electron transport length as a key descriptor of redox utilization in π-stacked organic crystals. Systematic pH-dependent results reveal a mechanistic transition in PDI carbonyl reduction from proton-coupled electron transfer to cation-mediated charge compensation, accompanied by a five-order-of-magnitude suppression in apparent rate constants. The anomalous pH dependence arises from kinetical asymmetry and proton-transport-limited cathodic activation. In contrast, TPA oxidation is governed by anion uptake, with activity strongly dependent on anion valence and coordination strength. Comparative studies with a less conjugated analogue further demonstrate that extended π-conjugation enhances both electronic accessibility and interfacial kinetics. By acquiring statistically robust kinetic data across large ensembles of individual particles, this single-particle framework delivers mechanistic resolution that is inaccessible to bulk electrochemistry. This work provides a clear molecular design roadmap for next-generation bipolar organic electrodes.

## Abstract 2

Single-particle electrochemical mapping of bipolar organic electrodes reveals sub-particle heterogeneity and elucidates ion-coupled electron-transfer kinetics in aqueous electrolytes.

## Graphical abstract

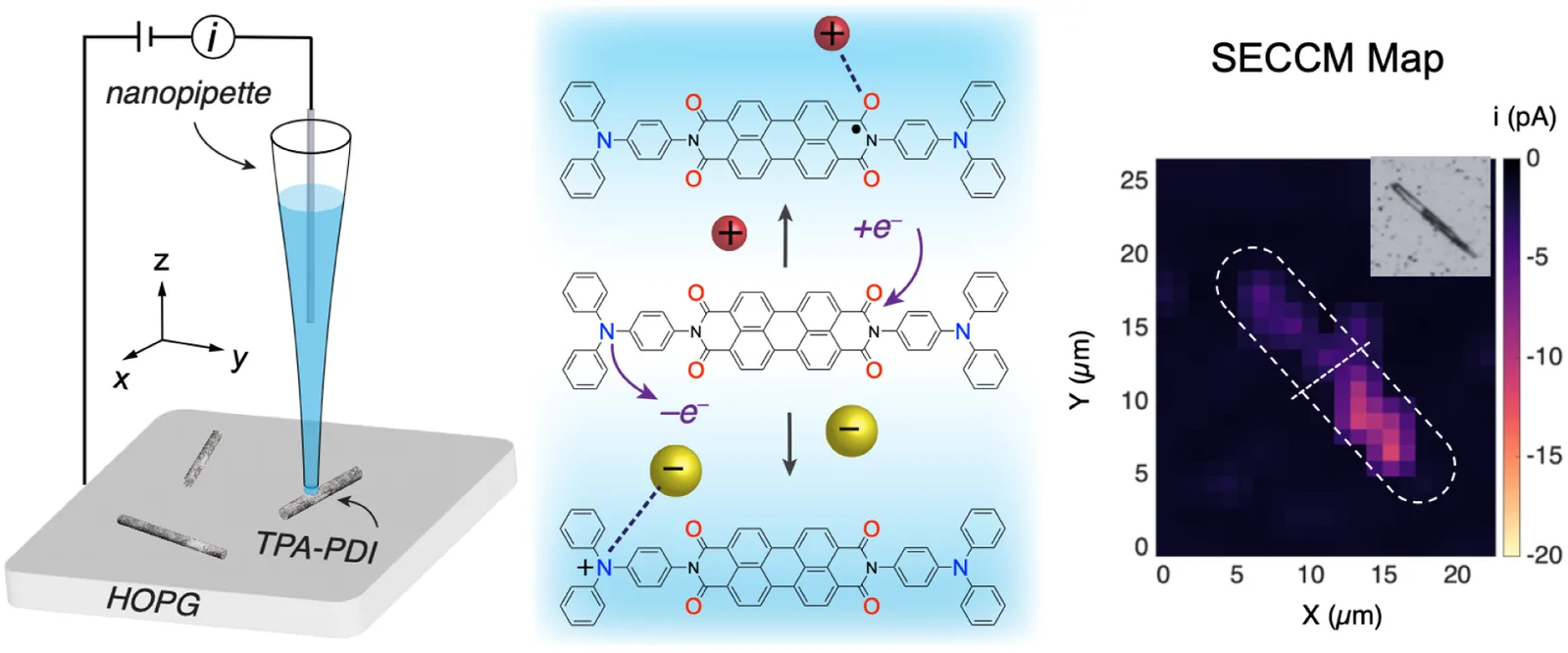

## Introduction

Organic electrode materials (OEMs) have emerged as promising alternatives to conventional transition metal oxides for electrochemical energy storage materials owing to their earth abundance and synthetic tunability.^1–3^ Unlike inorganic electrodes, whose charge-storage properties are largely dictated by fixed crystal structures and compositions, OEMs offer molecular-level control over redox thermodynamics, reaction kinetics, and ion interactions through rational structural design.^4,5^ The electrochemical response of OEMs stems from ion coupled electron transfer at molecular redox centers. This flexibility creates opportunities to engineer charge storage processes beyond conventional insertion chemistry. However, understanding the structure–reactivity relationship, particularly in aqueous environments, remains challenging because charge compensation pathways can depend sensitively on proton activity, ion identity, molecular packing, and local transport environments.

**Fig. 1:**
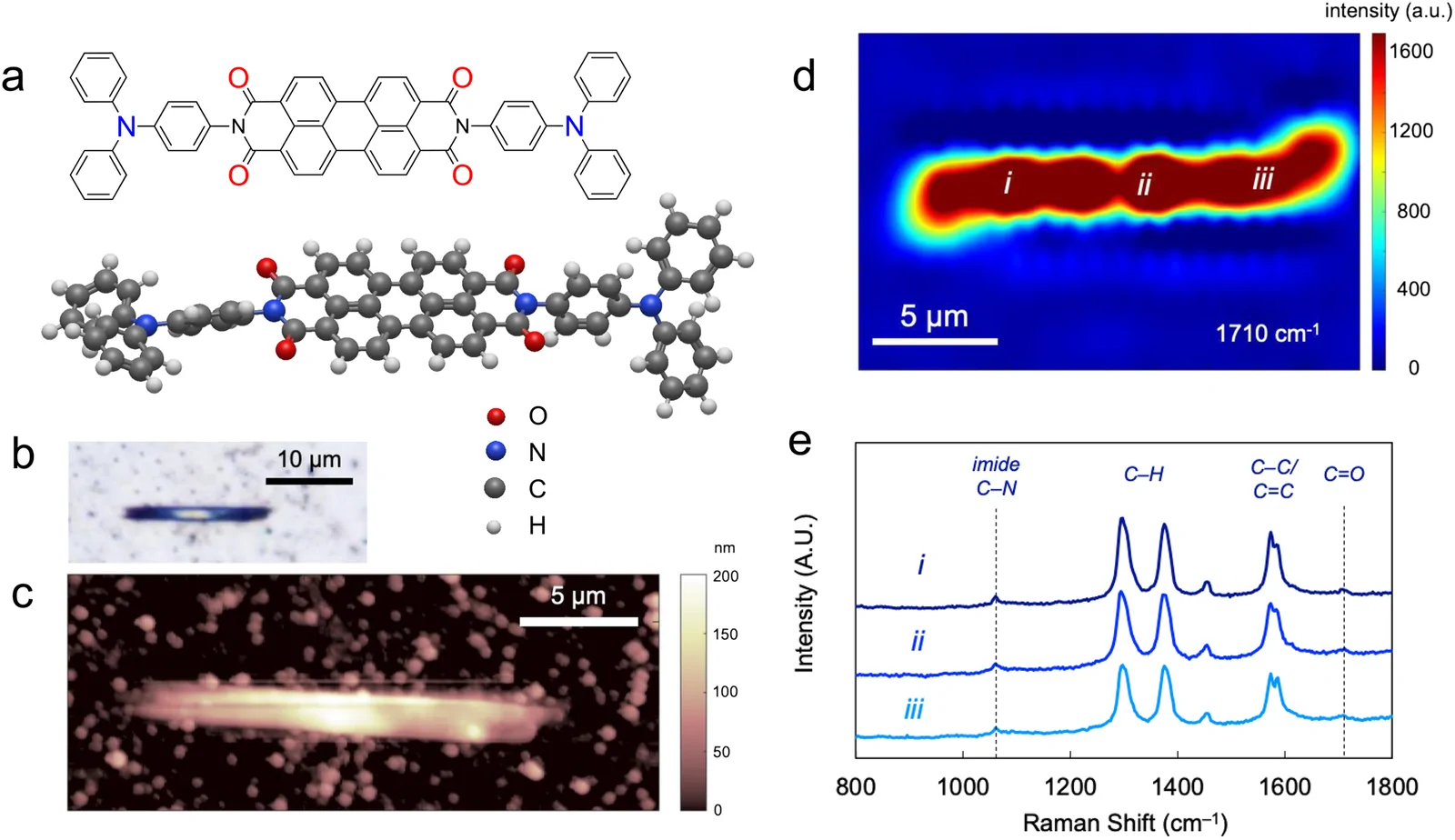
(a) Molecular structure of PDI–TPA. (b) Optical micrograph showing a PDI–TPA crystal drop-cast onto an HOPG substrate. (c) Non-contact AFM topographic image of the same PDI–TPA crystal shown in panel (b). (d) Raman scattering intensity map of the crystal in panel (b) at 1710 cm^−1^. (e) Local Raman spectra collected at different positions indicated in panel (d).

Among emerging OEM architectures, bipolar molecules that integrate electron-accepting (n-type) and electron-donating (p-type) motifs provide a powerful platform, as they combine multiple charge storage pathways within a single molecular framework.^6^ A broad range of bipolar systems, including conjugated polymers^7^ and molecular architectures incorporating quinones,^8–10^ imides,^11–14^ nitroxides,^15^ aromatic amines,^16,17^ and disulfides^18^ have demonstrated enhanced charge storage through complementary redox processes. Among them, molecular systems based on perylene diimide derivatives (PDI) and triphenylamine (TPA) offer a particularly well-defined and modular platform. PDI provides a chemically robust and structurally precise n-type unit with well-established carbonyl-centered redox chemistry,^19,20^ while TPA serves as a prototypical p-type moiety with reversible amine oxidation and tunable redox properties.^21,22^ As depicted in Fig. 1a, bipolar PDI–TPA presents a conjugated framework by covalent integration of these two units with controlled spatial proximity. These features make PDI–TPA an attractive model system for multifunctional charge storage,^23^ electrochromic,^24^ and photovoltaic^25^ applications.

The charge storage mechanisms and redox properties of PDI-derived and TPA-derived OEMs have been extensively investigated. For instance, PDI-based electrodes have shown to reversibly store Mg^2+^ ions with high stability and rate capability owing to hydrogen bond formation during ion insertion.^11^ The influence of Li^+^, Na^+^, and K^+^ on carbonyl-based OEMs has revealed that ion size and structure flexibility strongly modulate the thermodynamic and kinetic properties.^13^ Molecular twisting of the dihedral angle and substitution in PDI has been used to tune redox potentials and the shape of voltage profiles.^14^ In parallel, TPA-based frameworks have shown reversible amine oxidation, accompanied by the stabilization of radical cations through structural confinement.^17^ By integrating the two groups, we have demonstrated PDI–TPA as a promising bipolar cathode that exhibits high capacity and long cycle life.^23^

Beyond aprotic phases, aqueous electrolytes are increasingly attractive for battery applications due to their intrinsic safety, low cost, and high ionic conductivity.^26,27^ PDI-based OEMs have been explored with a range of charge carriers, including Zn^2+^,^28^ Li^+^,^29^ and Ca^2+^,^12^ demonstrating their versatility for water-based electrolytes. Despite substantial advancements, the proton availability and proton-coupled electron transfer (PCET)^30–32^ in the aqueous environment, which may modify reaction pathways and kinetics, remain underexplored. The electrolyte pH has shown significant effect on the cell performance of quinone-based lithium-ion batteries,^10^ however, the mechanistic interplay among proton transfer, counterion compensation, molecular structure, and local charge-transport kinetics remains insufficiently understood. This gap represents a critical bottleneck in the rational design of bipolar OEMs for aqueous battery applications.

A central challenge in resolving these mechanisms is that conventional electrochemical measurements probe the averaged behavior of a large quantity of electrode materials, which is obscured by the intrinsic heterogeneity of the ensembles. Variations in particle morphology, thickness, crystallinity, and electronic connectivity lead to strongly spatially nonuniform reactivity, making it difficult to extract intrinsic structure–reactivity relationships that remain hidden in bulk measurement.^33,34^ To overcome this limitation, we employ scanning electrochemical cell microscopy (SECCM)^33,35^ to conduct localized electrochemical interrogation and direct mapping of redox activity at the level of individual microscale particles. These spatially resolved, single-particle electrochemical approaches have effectively revealed the charge and discharge characteristics of various electrode materials including LiFePO_4_,^35,36^ LiCoO_2_,^37^ LiMn_2_O_4_,^38^ and graphene.^39,40^ Here, we use SECCM to probe the dual redox behavior of a bipolar PDI–TPA OEM in aqueous media, resolving spatial heterogeneity in both carbonyl- and amine-centered processes. By addressing these OEM particles individually, we reveal how particle thickness, electrolyte pH, counterions, and molecular structures govern the redox behavior of PDI–TPA systems in aqueous electrolytes. This single-entity perspective provides unique statistical and mechanistic insights that are inaccessible from ensemble measurements.

## Results and discussion

### Characterizing the heterogeneity of PDI–TPA

Our PDI–TPA OEM was synthesized *via* a one-pot condensation reaction reported previously, where the functional groups have been thoroughly characterized.^23^Fig. 1a shows the molecular structure of PDI–TPA. The molecule is built around a planar PDI core with two TPA arms project away from the molecular plane at dihedral angles imposed by steric interactions at the imide nitrogen. The overall bipolar architecture thus combines a conjugated, planar electron acceptor (PDI) with two separated peripheral electron donors (TPA). The X-ray diffraction pattern of PDI–TPA crystals (Fig. S1a) exhibits a larger number of diffraction peaks than the PDI core, suggesting that incorporation of the bulky TPA substituents leads to more complex molecular packing and reduced structural order. The distinct peaks confirm that PDI–TPA retains a semi-crystalline structure with preserved intermolecular π–π interactions. This partially disordered packing may facilitate ion accessibility while maintaining electronic connectivity for electrochemical charge storage.

Elongated, needle-like microcrystals of PDI–TPA were drop-cast onto a highly oriented pyrolytic graphite (HOPG) substrate (Fig. 1b). This morphology reflects the self-assemble of PDI derivatives through π–π stacking interactions along the long molecular axis. Under optical examination, notable contrast heterogeneity is visible within individual crystals: the central regions of each needle appear bright white, while the peripheral and end regions are considerably darker. AFM topographic imaging of the same crystal (Fig. 1c) reveals that this optical contrast is originated from the thickness variation within the same particle. The bright white central regions correspond to significantly thicker domains, with heights of approximately 200 nm, while the darker peripheral regions are considerably thinner (∼60 nm). Our AFM measurements of additional representative PDI–TPA particles (Fig. S2) demonstrate similar structural characteristics, with the bright regions measuring 216 ± 24 nm in thickness and the dark regions measuring 64 ± 9 nm in thickness.

To examine whether this structural heterogeneity is accompanied by variation in chemical composition across the particle, we collected the Raman intensity map at 1710 cm^−1^ of the same crystal shown in Fig. 1b and c. This wavenumber corresponds to the symmetric C=O stretching vibration of the imide groups in the PDI core.^41^Fig. 1d presents the Raman map, showing uniform intensity across the entire particle and no correlation with the thickness variations. This implies a homogeneous molecular coverage throughout the particle, which is further supported by the local Raman spectra collected at three representative positions. In Fig. 1e, all spectra are essentially identical in peak positions and relative intensities, confirming that the coverage of all other functional groups is also uniform throughout the crystal.

### SECCM mapping of local redox activity

**Fig. 2:**
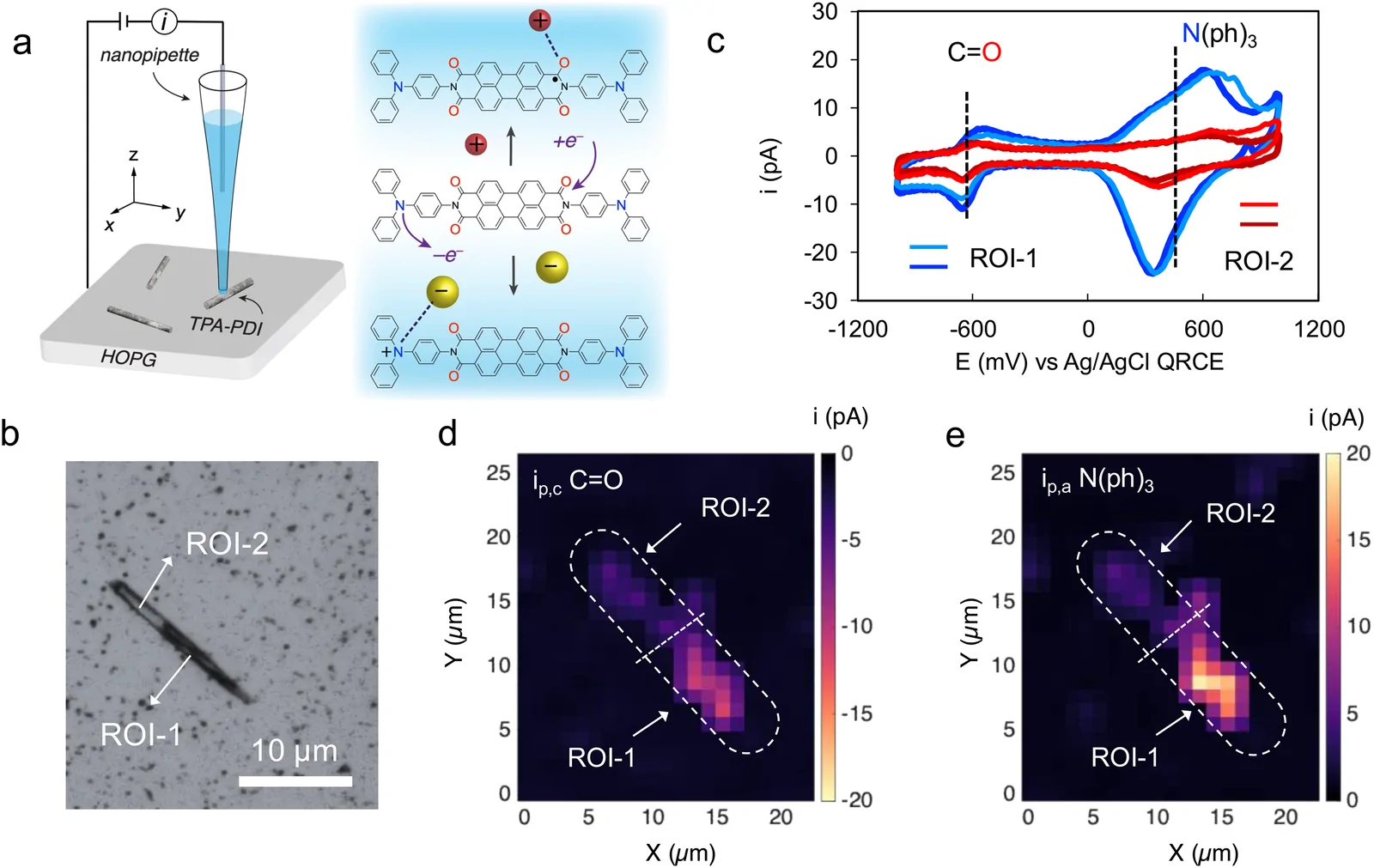
(a) Schematic illustration of an SECCM experiment on individual PDI–TPA particles. In the localized electrochemical nanocells, the reduction of PDI and oxidation of TPA are coupled with cation and anion transfer, respectively. (b) Optical image of a PDI–TPA particle highlighting ROI-1 and ROI-2. (c) Local voltammograms recorded at the ROIs indicated in panel (b) with a scan rate of 5 V s^−1^. The aqueous electrolyte contains pH 1.3 phosphate buffer and 0.45 M Na^+^. (d and e) SECCM current maps of the PDI–TPA particle collected at the carbonyl cathodic peak (d) and the amine anodic peak (e).

The observed heterogeneities motivate us to spatially resolve the electrochemical activity of individual PDI–TPA particles. In an SECCM experiment,^42^ a nanopipette containing an electrolyte solution and a Ag/AgCl quasi-reference counter electrode (QRCE) is brought into contact with the sample surface, forming a confined electrochemical cell at the point of contact (Fig. 2a). The nanopipettes used in this work are in the submicron range (SI Section 1.4) and well below the size of individual PDI–TPA particles. Local voltammetric measurements are performed within this nanoscale droplet cell, and the pipette is retracted and repositioned to an adjacent location, allowing a map of local electrochemical response to be created across the particle surface. This approach effectively captures the electrochemical response of distinct sub-particle regions, which would otherwise be averaged together in a conventional macroscale measurement. The right panel of Fig. 2a depicts the bipolar redox chemistry of PDI–TPA involving electron-transfer events coupled with distinct ion-transfer processes. The reduction of the PDI carbonyl groups proceeds *via* coordination of cations from the electrolyte to the imide oxygen, while oxidation of the TPA amine groups is charge-compensated by anion uptake from the solution.^23^

The optical image in Fig. 2b identifies two regions of interest (ROI-1 and ROI-2) within another representative PDI–TPA particle. Consistent with the example in Fig. 1b and c, ROI-1 corresponds to the optically darker, structurally thinner region, while ROI-2 corresponds the brighter, thicker domain. Under acidic buffer condition (pH 1.3), local voltammograms were collected by positioning the nanopipette probe at the two ROIs. The voltammograms in Fig. 2c display two pairs of redox peaks, confirming that both the PDI carbonyl and TPA amine redox centers are electrochemically active. The peak pairs around −0.6 V *vs.* Ag/AgCl QRCE and 0.5 V *vs.* Ag/AgCl QRCE are assigned to the chemically reversible but electrochemically quasireversible redox processes of the PDI carbonyl groups and TPA amine nitrogen, respectively.^23^ Fig. S3 presents repeated voltammetric measurements collected from a different representative PDI–TPA particle, demonstrating consistent redox responses across multiple scans and robust local stability during the charge/discharge process within the experimental timeframe.

Fig. 2c shows two voltammogram traces from different locations within ROI-1 (in blue) and two traces from different locations within ROI-2 (in red). The two voltammograms from the same ROI exhibit a high degree of similarity, indicating consistent and uniform redox activity within the domain. However, ROI-1 displays significantly higher currents for both carbonyl and amine redox peaks compared to ROI-2. This sub-particle electrochemical heterogeneity is further supported by the SECCM current maps shown in Fig. 2d and e, which were collected at the carbonyl cathodic peak potential (Fig. 2d) and the amine anodic peak potential (Fig. 2e), respectively. In both maps, the current distribution mirrors the topographic contrast: higher currents correspond to thinner regions and lower currents to thicker regions. Given the uniform molecular coverage characterized by Raman mapping (Fig. 1d and e), these results establish that PDI–TPA particles exhibit significant sub-particle electrochemical heterogeneity that is directly governed by local thickness. These findings are fully reproducible, as confirmed by additional SECCM maps presented in Fig. S4.

Despite containing less active material, the thinner domains exhibit enhanced current magnitude. This thickness-dependence points to charge transport within the organic crystal as a key factor governing electrochemical utilization. Notably, the peak positions, separations, and shapes across ROI-1 and ROI-2 (Fig. 2c) are nearly identical, with only the current magnitude differing. Specifically, ROI-1 shows peak separations of 92 mV and 264 mV for the PDI and TPA redox processes, respectively, while ROI-2 shows comparable peak separations of 97 mV and 268 mV for the corresponding PDI and TPA redox processes. This indicates that ionic diffusion is not the rate-limiting factor, since ion-transport-limited processes would manifest as peak broadening and potential shifts, which will be discussed later. Instead, electronic transport through the low-conductivity organic crystal governs the fraction of electrochemically accessible molecules. Electron transport in PDI–TPA relies on intermolecular hopping between adjacent π-stacked molecules rather than delocalized band-like conduction.^43^ In the thick domains represented by ROI-2, the redox centers in contact with electrolyte are electronically disconnected from the HOPG current collector due to a significant hopping distance, leaving a substantial fraction of the molecules electrochemically inactive. In thinner regions (ROI-1), the particle thickness is within the electronic penetration depth, enabling more complete electrochemical utilization of the active material. We also note that the currents measured in SECCM are very small, so electronic resistance is not expected to generate an *iR* drop large enough to significantly increase the peak separation. These findings highlight that engineering thinner particles with dimensions commensurate with the electronic penetration depth is more effective than simply increasing redox-site density.

### pH dependent bipolar redox behavior

To elucidate the effect of proton availability on the thermodynamics and kinetics of the PDI–TPA electrode, we systematically vary the pH of the phosphate buffer and employ our single particle approach to investigate the electrochemical response. All single-particle SECCM voltammograms presented were acquired in the dark, thinner regions (ROI-1) of PDI–TPA particles, where electron transport limitations are minimized, thereby providing an accurate representation of the intrinsic molecular redox behavior.

**Fig. 3:**
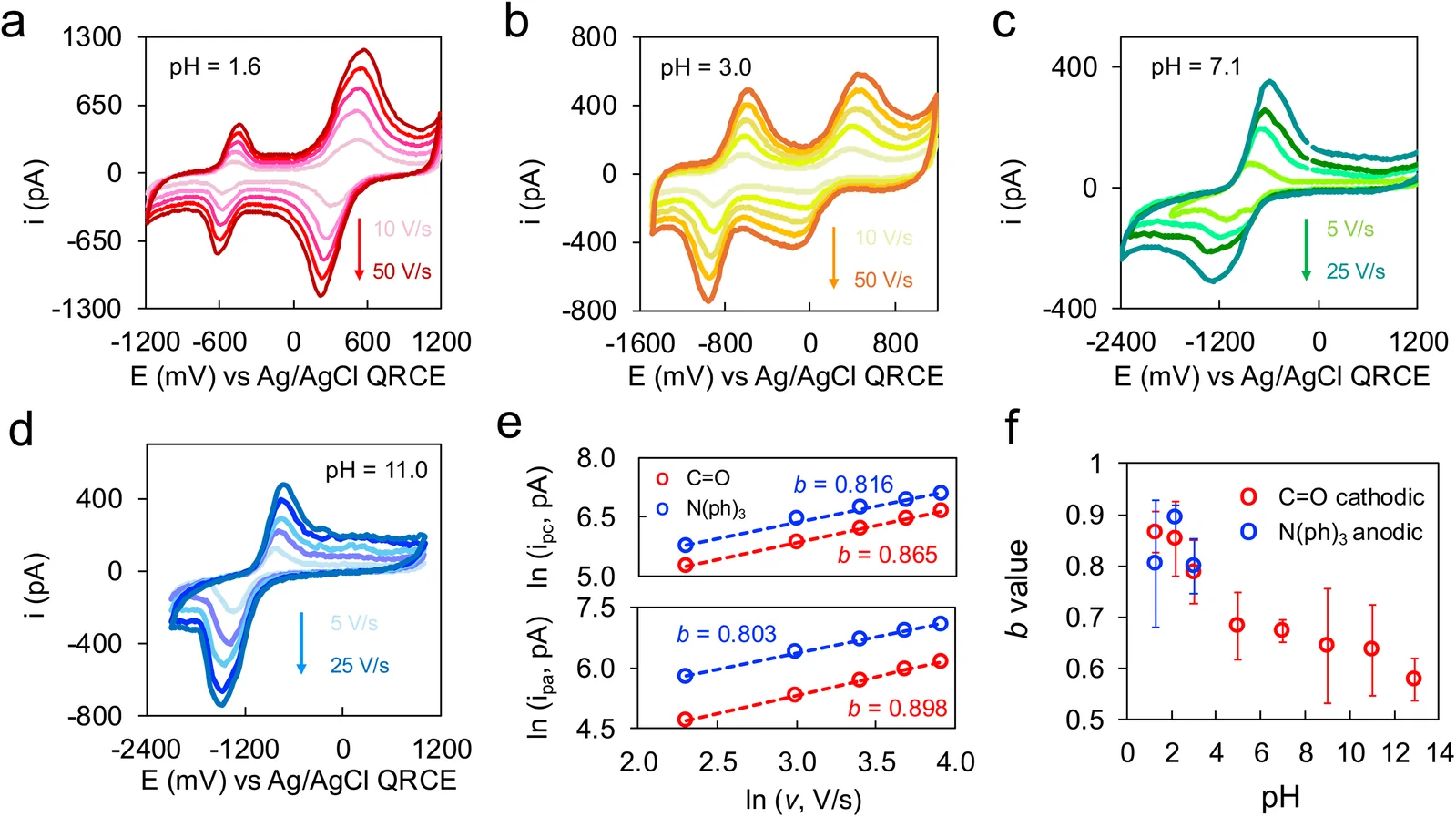
(a–d) Representative local voltammograms recorded at various scan rates in phosphate buffers containing 0.50 M Na^+^ at pH 1.6 (a), 3.0 (b), 7.1 (c), and 11.0 (d). (e) Plots of the natural logarithm of cathodic peak current (upper panel) and anodic peak current (lower panel) *versus* the natural logarithm of the scan rate for PDI (red) and TPA (blue) in pH 1.6 electrolyte. (f) pH-dependent *b* values derived from ln(*i*_pc_) *vs.* ln(*v*) (PDI, red) and ln(*i*_pa_) *vs.* ln(*v*) (TPA, blue).

We selected four pH conditions from our full pH spectrum data to show representative voltammograms recorded at various scan rates, as displayed in Fig. 3a–d. Fig. S5 shows representative voltammograms obtained at other pH conditions. At pH 1.6 (Fig. 3a), the peak currents for both redox couples increase systematically with increasing scan rate, as expected for electrochemically quasi-reversible process. The two-electron nature of full PDI reduction and TPA oxidation (two TPA groups, one-electron transfer each) is both resolved as single peaks. The anodic peaks associated with TPA oxidation are higher than the cathodic peaks of PDI reduction. This difference is likely due to structural factors: the peripheral TPA arms are more accessible to the electrolyte, while the PDI core is more deeply embedded within the π-stacking crystal and only partially ion-accessible.

Upon increasing the pH to 3.0 (Fig. 3b), the carbonyl reduction peak shifts to a clearly more negative potential, consistent with the pH-dependent thermodynamics of a proton-coupled electron transfer (PCET) process at the PDI center. This pH dependence will be further quantified and analyzed in the subsequent section. Additionally, the peak separation of the TPA amine redox increased dramatically, indicating a significant slowdown in the apparent kinetics of the amine oxidation/re-reduction process.

At pH 7.1 (Fig. 3c), the voltammogram exhibits a complete absence of amine redox activity, with no detectable TPA redox peaks within the accessible potential window. The PDI carbonyl couple remains present but with substantially increased peak separation, indicating substantially slower cation transport kinetics during the carbonyl process. At pH 11.0 (Fig. 3d), the electrochemical behavior closely resembles that at pH 7.1. TPA activity remains absent, and the carbonyl peaks present with large peak separation. These pH-dependent electrochemical behaviors highlight the key role of proton environment for both redox processes.

To quantify how charge storage mechanism changes with pH, we analyzed the dependence of peak current on scan rate using the power-law relationship (eqn (1)):

1 *i*_p_ = *av*^*b*^

where *i*_p_ is the peak current, *a* is a constant, *v* is the scan rate, and *b* is the diagnostic exponent that can be readily extracted from the slope of linear ln(*i*_p_) *vs.* ln(*v*) plot. *b* = 1 indicates a surface-confined process in which all electroactive material participates simultaneously, while *b* = 0.5 indicates a semi-infinite diffusion-limited process in which the overall rate is governed by ion transport. Intermediate values between 0.5 and 1 indicate mixed behavior.^1^ Representative ln(*i*_p_) *vs.* ln(*v*) plots for the PDI and TPA redox peak at pH 1.6 shown in Fig. 3e exhibit well-defined linear fits. At this pH, the observed *b* values of 0.8–0.9 for both reactions suggest behavior that is predominantly surface-confined.

This analysis extends across the pH range (1.6–13) using our single-particle approaches. Fig. 3f summarizes the mean *b* values for PDI and TPA processes, with error bars indicating standard deviations across multiple particles. As pH increases, *b* decreases systematically for the PDI carbonyl process, falling from 0.8–0.9 at strongly acidic pH to approximately 0.6 at pH 13. At low pH, the high proton concentration in the electrolyte ensures rapid cation transport into the PDI crystal. As pH increases and proton availability diminishes, the ion transfer slows down and transitions to a different mechanism. The *b* values (>0.8) for the TPA redox process could only be extracted at pH < 3, showing efficient anion transport in acidic conditions. The pH effects on both redox behaviors are discussed separately below.

### Mechanism and kinetics of PDI redox

**Fig. 4:**
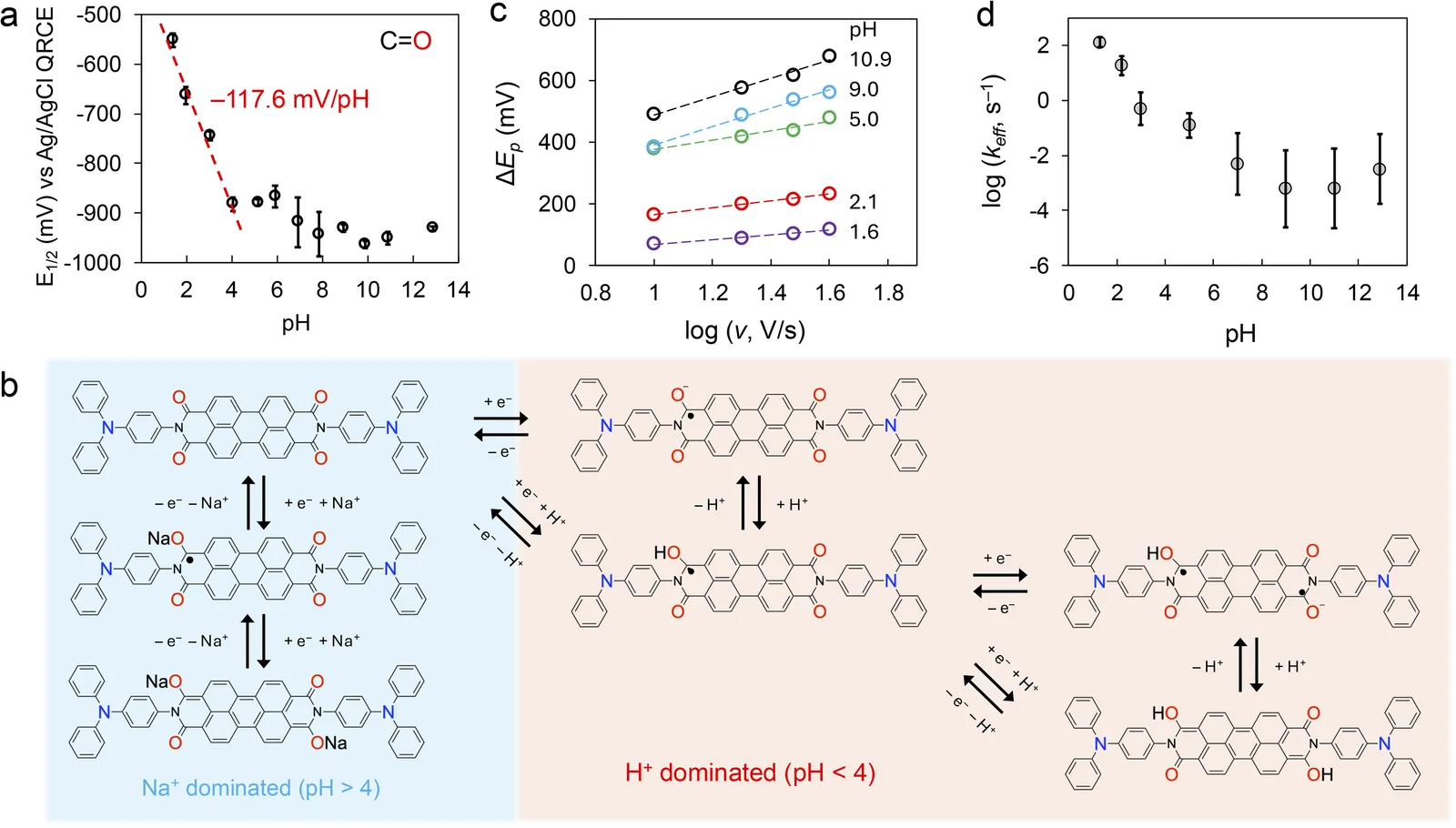
(a) Plot of half wave potential, *E*_1/2_, of PDI carbonyl redox as a function of pH. A slope of 117.6 mV per pH unit is observed for pH < 4. (b) Schematic illustration of the PDI redox reaction mechanism in the proton-rich regime (in red) and the proton-deficient regime (in blue). (c) Plot of peak separation, Δ*E*_p_, *versus* logarithm of the scan rate under selected representative pH conditions. (d) Plot of the logarithm of the effective rate constant, *k*_eff_, as a function of pH.

The PDI carbonyl center undergoes a well-established two-electron reduction,^19,20^ proceeding through two sequential one-electron steps with charge compensation provided by cations from the electrolyte. We determined the half-wave potentials (*E*_1/2_) by taking the midpoint between the cathodic and anodic peaks of the PDI reaction from all SECCM voltammograms, which is conventionally considered as the thermodynamic formal potential (*E*°′). In Fig. 4a, the mean *E*_1/2_ value shifts linearly with pH at a slope of 117.6 mV per pH unit below pH 4, substantially exceeding the Nernstian expectation of 59 mV per pH for a 2H^+^/2e^−^ stoichiometry.^31^ Intuitively, this apparent super-Nernstian behavior suggests a 2H^+^/1e^−^ proton-coupled electron transfer process, as predicted from the Nernst relationship (eqn (2)):2
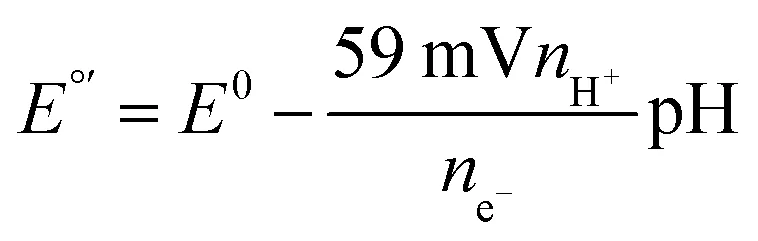
where *n*_H^+^_/*n*_e^−^_ = 1.99 denotes the number of protons per electron transfer. However, the experimentally measured *E*_1/2_ should not be treated as directly equivalent to *E*°′. The 2H^+^/1e^−^ mechanism is difficult to justify structurally in the present PDI–TPA system. Although additional protonation at either the imide or TPA nitrogen could theoretically provide an additional proton source, both are weakly basic due to strong resonance stabilization.^44^ Such scenarios are therefore unlikely under the mildly acidic conditions in this study. The apparent super-Nernstian slope may instead reflect a combination of kinetic and ion-transport effects associated with charge compensation during the cathodic process, with proton transport representing one possible contribution. As shown in Fig. S6, the cathodic and anodic peak potentials each exhibit distinct slopes against pH, with the cathodic peak shifting more steeply than the anodic. This asymmetry suggests that the reduction process is limited by the proton transport into the active materials, pushing the cathodic peak more negative and inflating the *E*_1/2_ slope. The reversible oxidation appears less affected by proton transport. Such behavior suggests an effective charge transfer coefficient (*α*) of less than 0.5 and an asymmetric activation barrier that requires larger overpotentials to drive the cathodic process. Above pH 4, *E*_1/2_ remains largely constant and shows only minimal variation with increasing pH, indicating a proton-independent pathway as proton availability diminishes.

This pH effect defines two mechanistic regimes illustrated in Fig. 4b. In the proton-rich regime (pH < 4, shown in red), the elementary steps of the PDI reduction are depicted with either concerted or stepwise PCET pathways. In the concerted pathway (diagonal arrows), electron and proton transfer occur simultaneously in a single step; while in the stepwise pathway (horizontal and vertical arrows), electron and proton transfer proceed sequentially through discrete ˙C–O^−^ radical intermediates.^8,20^ In the proton-deficient regime (pH > 4, shown in blue), proton transfer is nearly absent and Na^+^ assumes the charge-compensation role. The switch in charge-compensating ion has a direct consequence for the charge storage dynamics revealed in Fig. 3f. Protons have greater mobility compared to Na^+^ ions, allowing for faster penetration and enhanced utilization of the carbonyl sites, which aligns with the near-surface-confined behavior observed at low pH (*b* ≈ 0.9). As Na^+^ becomes the dominant charge carrier above pH 4, its slower transport drives the transition toward diffusion-limited behavior (*b* ≈ 0.6).

Since the redox centers are bound on the PDI–TPA electrode, we quantify the electrochemical kinetics by analyzing the peak potentials as a function of scan rate based on the Laviron method.^45^ It is worth noting that the derived parameters at higher pHs, where the response transitions from surface-confined toward cation-transport-limited behavior, should be regarded as apparent kinetic descriptors rather than a complete description of the underlying processes. Fig. 4c plots the peak separation (Δ*E*_p_) against log(*ν*) for several representative pH conditions. As pH increases, Δ*E*_p_ becomes larger, indicating greater irreversibility and slower kinetics. Moreover, the slope of Δ*E*_p_*versus* log(*ν*) also shows a strong dependence on pH, increasing from ∼100 mV per decade to ∼280 mV per decade as pH increases. While the slope is dependent on *α* and the number of electron transfer (*n*) in the Laviron model, we could not reliably extract the *α* values due to their potential dependent nature in a PCET process.^46^ Nevertheless, the larger slope indicates an increased kinetic asymmetry (*α* ≠ 0.5, consistent with Fig. 4a) and decreased *n* as proton availability decreases. In this context, a reduced effective *n* does not imply a change in the intrinsic redox stoichiometry but rather reflects increased kinetic barriers on proton delivery and limited access to carbonyl redox sites.

Because the values of *α* and *n* are not reliably accessible using the Laviron method, we evaluate the reaction kinetics using an effective rate constant, *k*_eff_, as described in eqn (3):3
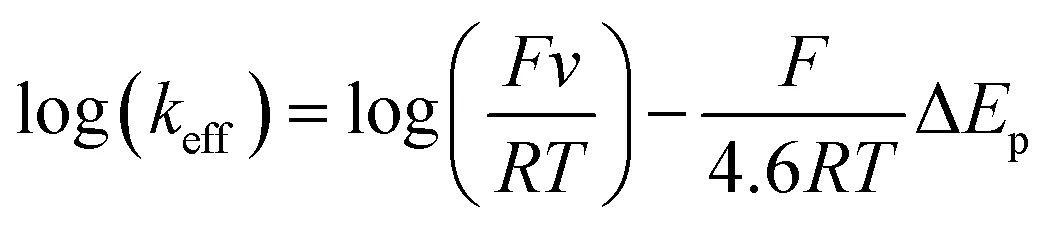


It is important to note that the extracted *k*_eff_ solely reflects the apparent electrochemical kinetics observed in voltametric responses, rather than the intrinsic electron transfer rate constant. At a fixed scan rate, a larger Δ*E*_p_ corresponds to a smaller *k*_eff_. Individual *k*_eff_ values extracted from this analysis are shown in Fig. S7. Fig. 4d presents a plot of the mean values of log(*k*_eff_) for each pH with standard deviations represented as error bars. This trend shows a monotonically decreasing dependence on pH with a shallow plateau in the alkaline regime. The *k*_eff_ value drops by nearly five orders of magnitude from pH 1 to 11, due to the attenuation of the proton-assisted PDI reduction by decreased H_3_O^+^ activity. Notably, a slight recovery in *k*_eff_ is observed at pH 13, consistent with the emergence of a water/hydroxide-mediated PCET pathway, as reported for surface-confined carboxylate sites.^47^ However, this recovery is limited because the PDI imide carbonyl cannot effectively engage water as a proton donor.

We further infer the PCET mechanism from the strong pH dependence of *k*_eff_ in the acidic regime. A stepwise mechanism, in which electron transfer is decoupled from proton transfer, would yield pH-independent electrode kinetics. Instead, the observed exponential sensitivity of *k*_eff_ to pH (Fig. 4d) suggests a concerted PCET process, where both transfer events occur in a single kinetic step and the overall rate is directly gated by proton availability. Above pH 4, as Na^+^ replaces protons as the dominant charge-compensating cation, *k*_eff_ gradually transitions to a pH-independent plateau, confirming that proton activity no longer governs the electrode kinetics in the proton-decoupled regime.

Further quantitative insight can be extracted following the analytical framework developed by Lewis *et al.*^47^ The slope of log(*k*_eff_) *versus* pH dependence in the acidic regime yields an effective charge transfer coefficient *α* = 0.27 (see SI Section 3), consistent with our previous discussion. The proton transfer to the PDI imide carbonyl faces a substantial kinetic barrier and is strongly dependent on proton availability. Extrapolation of the linear fit to pH = 0 yields a standard rate constant ∼500 s^−1^, indicating a rapid intrinsic electron transfer rate at sufficiently high proton activity.

### Anion effects on TPA redox

Unlike the PDI carbonyl redox chemistry, which is primarily governed by proton and cation activity, the TPA moiety undergoes a p-type oxidation process in which an electron is removed from the triphenylamine nitrogen, N(ph)_3_, to generate a delocalized amine radical cation, (ph)_3_N˙^+^.^48^ The positive charge formed on the oxidized TPA unit is compensated by insertion of electrolyte anions into the organic particle to maintain electroneutrality. To systematically examine the role of electrolyte anions, we performed SECCM-based single-particle measurements in electrolytes that contain sulfate or perchlorate anions, in addition to the phosphate electrolyte discussed previously. Each electrolyte contained 0.50 M Na^+^, with the pH adjusted to either acidic or neutral conditions using the corresponding acid.

**Fig. 5:**
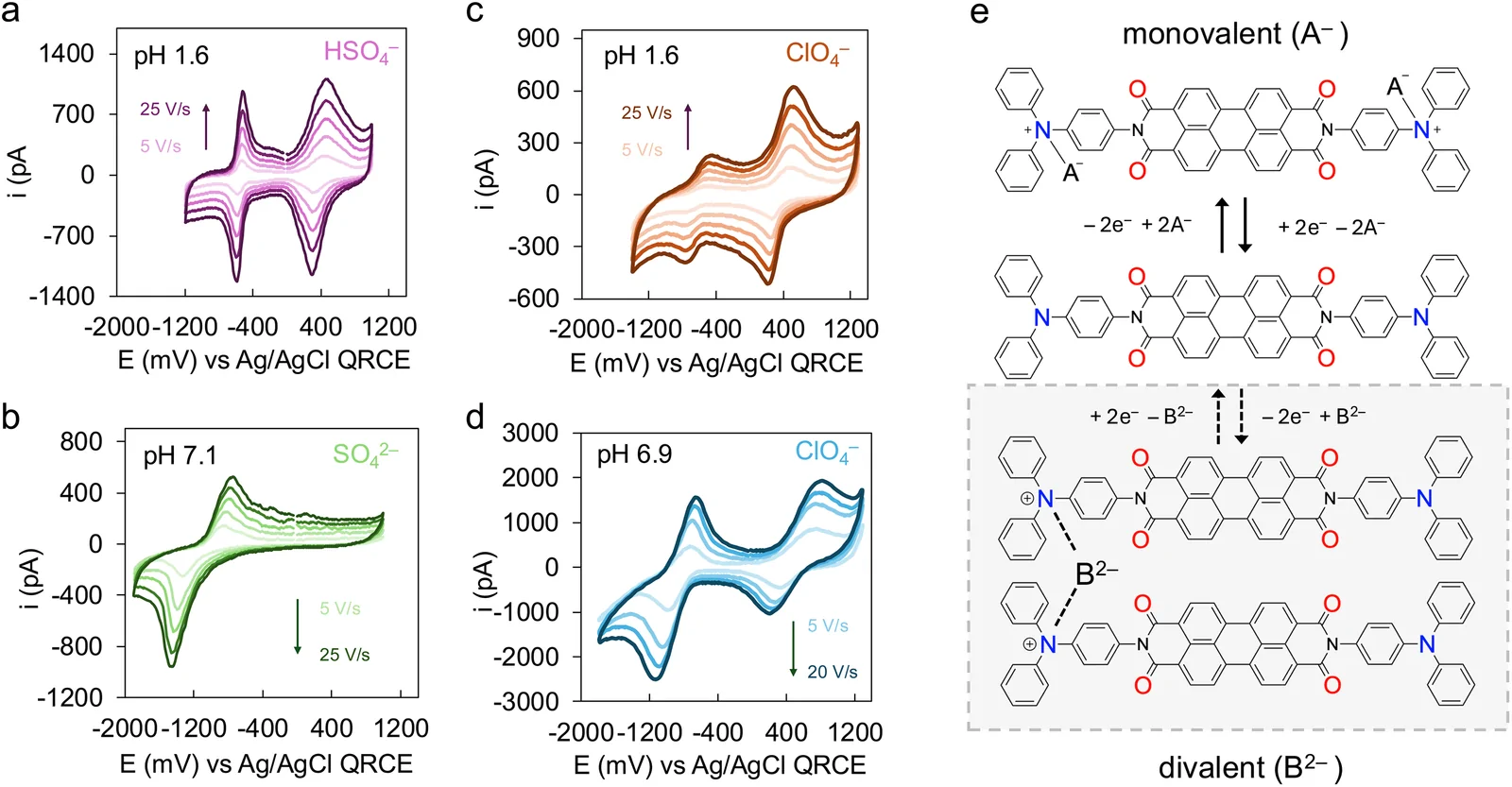
(a and b) Representative local voltammograms at various scan rates in sulfate electrolyte containing 0.5 M Na^+^ at pH 1.6 (a) and 7.1 (b). (c and d) Representative local voltammograms in perchlorate electrolyte containing 0.5 M Na^+^ at pH 1.6 (c) and 6.9 (d). (e) Schematic illustration of the TPA redox mechanism in the monovalent-anion regime (top) and in the divalent-anion regime (bottom).

Representative voltammograms obtained with sulfate and perchlorate electrolytes are shown in Fig. 5a–d. The response of phosphate is displayed in Fig. 3. All three anions in acidic conditions (pH < 2) support well-defined, nearly reversible TPA redox peaks (Fig. 5a, c and 3a). The behaviors change dramatically at neutral pHs. In sulfate and phosphate electrolytes, only PDI redox peaks are observable in Fig. 5b and 3c, while TPA activity vanishes entirely. Perchlorate continues to support well-defined TPA redox peaks at pH ∼7 (Fig. 5d), albeit with larger peak separations and slower kinetics.

This anion and pH dependent TPA activity is rationalized by the mechanistic schematic in Fig. 5e, which identifies two regimes governed by anion charge. In the monovalent anion regime (top), each N˙^+^ radical is independently charge-compensated by a monovalent anion (A^−^) in a 1 : 1 stoichiometry, providing a geometrically simple and energetically favorable insertion pathway. In the divalent anion regime (bottom), a divalent anion (B^2−^) cannot neutralize a single positively charged nitrogen on its own, and it must bridge between two N˙^+^ sites across adjacent molecules, imposing geometric and energetic constraints that significantly suppress TPA activity. At acidic pHs, sulfate and phosphate mainly exist in the monovalent protonated forms (HSO_4_^−^ and H_2_PO_4_^−^) and effectively facilitate TPA oxidation. As pH approaches neutral, they convert to divalent deprotonated forms (SO_4_^2−^ and HPO_4_^2−^) and fail to stabilize N˙^+^. Perchlorate (ClO_4_^−^) remains monovalent across the full pH range. In essence, the valence charge dictates the TPA oxidation behavior, while pH acts as a tuning knob that controls the charge state of multivalent ions, thereby modulating the TPA reactivity.

Perchlorate sustains active TPA oxidation in both acidic and neutral environment. However, its kinetic behavior reveals additional mechanistic nuance. Fig. S8 shows the distribution of individual log(*k*_eff_) values extracted from single particle voltammograms. Under pH ∼ 1.6, log(*k*_eff_, s^−1^) for TPA redox with sulfate, phosphate, and perchlorate are 1.79 ± 0.33, 1.09 ± 0.44, and 0.08 ± 0.31, respectively. HSO_4_^−^ and H_2_PO_4_^−^ are strong hydrogen bond acceptors that can stabilize the inserted state and lowering the kinetic barrier for anion uptake. In contrast, ClO_4_^−^ is a weakly coordinating anion with negligible hydrogen bonding ability,^49^ resulting in slower apparent kinetics of TPA reaction. Interestingly, the influence of anion identity also extends to the PDI carbonyl activity. In comparison to sulfate and phosphate, perchlorate exhibits larger peak separation (Fig. 5c). The more extensive hydrogen-bond networks formed by HSO_4_^−^ and H_2_PO_4_^−^ may facilitate local proton transfer, whereas ClO_4_^−^ is non-buffering and relies on proton diffusion from solution, leading to slower carbonyl kinetics.

At neutral pH, TPA activity is preserved in ClO_4_^−^ but log (*k*_eff_, s^−1^) drops to −1.96 ± 0.53. The TPA oxidation is typically regarded as a pure electron-transfer process without involvement of proton transfer.^48^ The decrease in reaction kinetics at higher pH may be attributed to changes in the local electrostatic environment rather than a PCET effect. We speculate that under neutral/basic conditions, the PDI core carries a partial negative charge that electrostatically repels ClO_4_^−^ anions from the OEM interface, thereby diminishing the apparent reaction rate.

### Effects of molecular conjugation

**Fig. 6:**
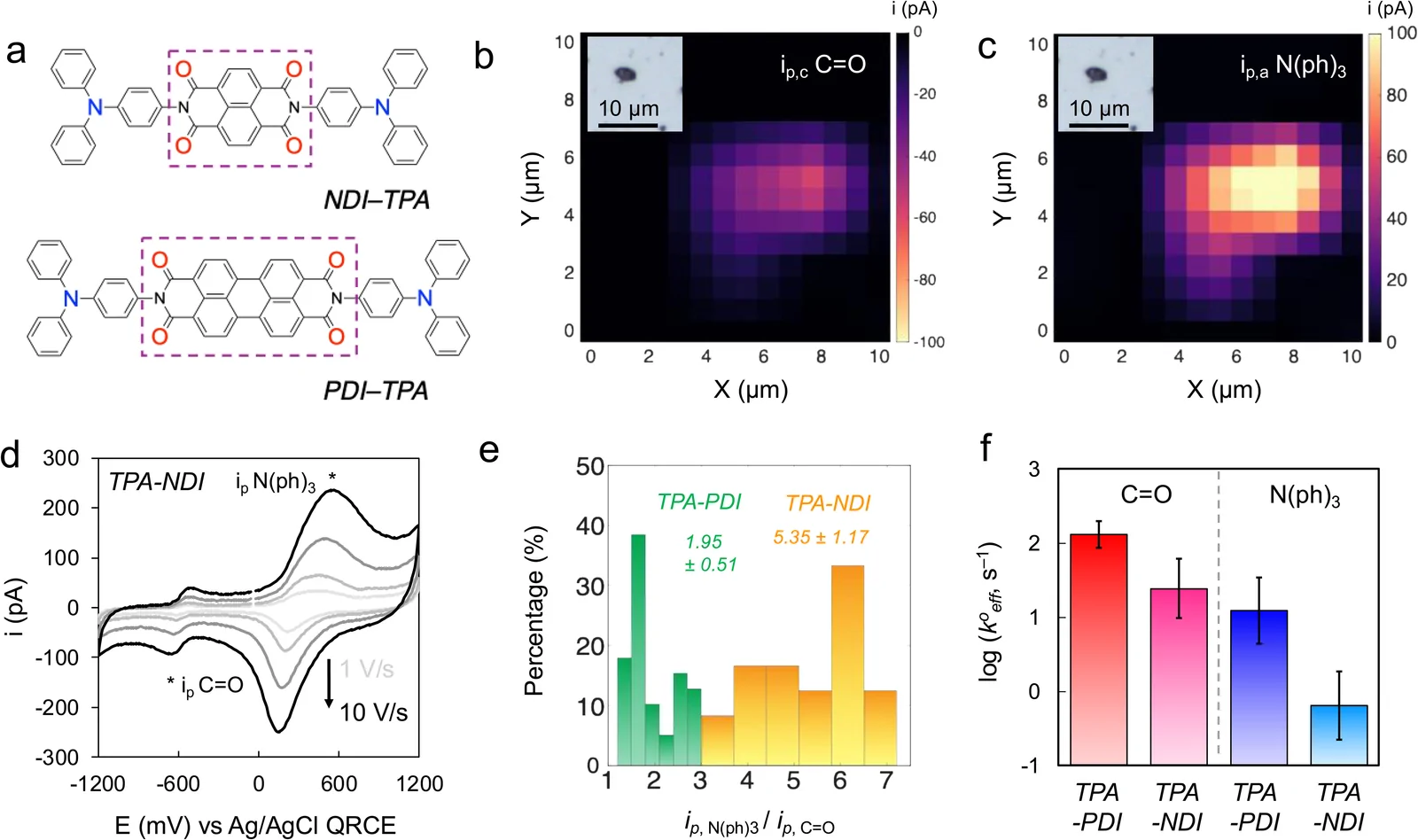
(a) Molecular structure of NDI–TPA compared to PDI–TPA. (b and c) SECCM current maps of the NDI–TPA particle collected at the carbonyl cathodic peak (b) and the amine anodic peak (c). (d) Representative local voltammograms recorded at NDI–TPA in phosphate buffers containing 0.5 M Na^+^ at pH 1.6. (e) Histograms of the ratio of amine anodic peak current to carbonyl cathodic peak current for NDI–TPA and PDI–TPA electrodes. (f) Comparison of the carbonyl and amine kinetics in NDI–TPA and PDI–TPA.

To investigate the effects of π-conjugation on the bipolar redox activity of OEMs, we compare PDI–TPA with its structural analogue NDI–TPA, where the perylene diimide core is substituted by a naphthalene diimide (NDI) (Fig. 6a). NDI contains only two fused aromatic rings, in contrast to the four rings in PDI, resulting in a significantly less conjugated and smaller π-system. The X-ray diffraction pattern of NDI–TPA qualitatively resemble that of PDI–TPA (Fig. S1b). Notably, the peak at 2*θ* = 25.1° observed in PDI–TPA shifts to 24.1° in NDI–TPA, indicating an increase in the spacing between molecular planes and a weakening of intermolecular π–π stacking interactions.^50^ NDI–TPA retains the same carbonyl and TPA components, making it a well-defined control for studying the impact of π-conjugation on electrochemical behavior.

SECCM current maps collected at the carbonyl cathodic peak (Fig. 6b) and the amine anodic peak (Fig. 6c) reveal reasonably uniform redox activity across the NDI–TPA particle. The NDI–TPA crystals show a spherical-like morphology compared to the elongated form of PDI–TPA. This difference is likely attributed to the weaker anisotropic π-stacking driving force in the less conjugated NDI–TPA system. The uniform activity maps allow us to capture the kinetic properties of NDI–TPA without being affected by morphological heterogeneity.

Representative local voltammograms recorded on NDI–TPA in acidic phosphate buffer solution are shown in Fig. 6d. The overall voltammogram shape is qualitatively similar to that of PDI–TPA, with two pairs of redox peaks assigned to the carbonyl reduction and amine oxidation processes respectively. However, compared with PDI–TPA (Fig. 3a), the cathodic peak associated with carbonyl reduction is substantially smaller relative to the anodic TPA oxidation peak. To quantify this difference, we performed single-particle statistical analysis across all particles measured under this condition. Histograms of the ratio of amine anodic peak current to carbonyl cathodic peak current (*i*_p,N(ph)_3__/*i*_p,C=O_) are shown in Fig. 6e. NDI–TPA exhibits a mean ratio of 5.35, significantly larger than the value of 1.95 observed for PDI–TPA. Since the TPA arms are identical in both molecules, this elevated ratio indicates a more pronounced reduction in the population of carbonyl sites that are reacted. This behavior is likely due to reduced conjugation of the NDI core. The larger perylene framework in PDI can better delocalize and stabilize the added electron density^51^ during carbonyl reduction, facilitating electron uptake at the carbonyl sites. In contrast, the less conjugated NDI core makes the ˙C–O^−^ intermediate less stabilized, resulting in suppressed carbonyl reduction currents in NDI–TPA. Weaker intermolecular packing in NDI–TPA may further limit charge transport and reduce the electrochemical accessibility of the carbonyl sites.

The comparison of apparent kinetics in acidic condition between NDI–TPA and PDI–TPA is shown in Fig. 6f. Both the carbonyl and amine processes exhibit higher *k*_eff_ in PDI–TPA relative to NDI–TPA. This is consistent with extended π-conjugation facilitating fast ionic transport *via* the formation of conduction channel.^50,52^ While the effects of conjugation on OEM performance have been recognized in ensemble studies, our single-particle SECCM approach isolates the intrinsic molecular-level response from particle-to-particle morphological variability and electrode fabrication artifacts, enabling a more direct and quantitative correlation between kinetics and molecular structure.

## Conclusions

This work demonstrates, for the first time, the direct mechanistic investigation of the redox chemistry within a bipolar OEM at the single-particle level. Our SECCM approach reveals the sub-particle heterogeneities associated with the crystal thickness that governs the electrochemical activity through electron transport across the low-conductivity organic crystal. The PDI carbonyl center undergoes concerted PCET, directly evidenced by the exponential sensitivity of the apparent rate to proton activity, highlighting the critical role of proton transfer in both the thermodynamics and kinetics of carbonyl reduction. TPA amine oxidation is jointly governed by electrolyte anion identity and pH, where the interplay between anion valence and protonation state determines the amine activity. Extended π-conjugation enhances the performance of both redox centers through improved ion transport and intermediate stabilization. Importantly, by analyzing electrochemical data across large ensembles of individual particles, this approach yields statistically robust results that capture the true variability of intrinsic reactivity. These findings establish single-particle approach as an indispensable tool for decoding the molecular-level complexity of bifunctional organic electrode systems.

These insights carry direct implications for aqueous organic battery development and for the broader concept of ion-coupled electron transfer in localized electrochemical systems. While prior droplet-confined electrochemistry studies^53–57^ have elucidated ion transfer across liquid–liquid interfaces, our work extends this concept to solid organic electrode particles. The concerted PCET mechanism at the PDI center and rapid kinetics at high proton activity demonstrate the promising potential of PDI-based OEMs as fast-charging cathode materials for proton batteries. The demonstration that anion valence can selectively switch TPA activity on or off highlights electrolyte anions as a crucial design variable. Extending this single-particle framework to a broader library of OEM architectures will bridge mechanistic understanding with macroscale device performance and accelerate the rational development of next-generation organic energy storage systems.

## Author contributions

Y. Y. and C. L. conceived the study. G. A. A., J. R. C., P. A., A. B. B., T. S., and V. W. conducted the experiments. G. A. A., J. R. C., and Y. Y. analyzed the data. Y. Y. drafted the manuscript and oversaw the study.

## Conflicts of interest

The authors declare no conflict of interest.

## Supplementary Material

SC-OLF-D6SC04619C-s001

## Data Availability

The data supporting this article have been included as part of the supplementary information (SI). Supplementary information: experimental methods, XRD data, additional AFM and SECCM maps, voltammograms with multiple scans, scan rate dependence of cathodic and anodic peaks, individual effective rate constants, SECCM probe characterization, potential stability test, and determination of the charge transfer coefficient. See DOI: https://doi.org/10.1039/d6sc04619c.

## References

[cit1] Gannett C. N., Melecio-Zambrano L., Theibault M. J., Peterson B. M., Fors B. P., Abruña H. D. (2021). Mater. Rep.: Energy.

[cit2] Lee S., Hong J., Kang K. (2020). Adv. Energy Mater..

[cit3] Li Z., Tang H., Liang Y., Liu Y., Li M., Ma L., Chen H., Zhai X., Wei X., Gu M. D., Wang J., Wang Y., Tong S., Jiang Q., Geng Y., Ma Y., Cao Y., Xu Y., Huang F. (2026). Nature.

[cit4] Holguin K., Mohammadiroudbari M., Qin K. Q., Luo C. (2021). J. Mater. Chem. A.

[cit5] Jia L., Bao P., Fan Z., Liu Y., Yao S., Xu Y. (2025). J. Mater. Chem. A.

[cit6] Tong Y., Wei Y., Song A., Ma Y., Yang J. (2024). ChemSusChem.

[cit7] Chen X., Zhang W., Zhang C., Guo Y., Yu A., Mei S., Yao C.-J. (2024). Adv. Sci..

[cit8] Yan L., Zhao C., Sha Y., Li Z., Liu T., Ling M., Zhou S., Liang C. (2020). Nano Energy.

[cit9] Han C., Li H., Shi R., Zhang T., Tong J., Li J., Li B. (2019). J. Mater. Chem. A.

[cit10] Jing Y., Liang Y., Gheytani S., Yao Y. (2020). ChemSusChem.

[cit11] Lei X., Zheng Y., Zhang F., Wang Y., Tang Y. (2020). Energy Storage Mater..

[cit12] Sahoo P., Goudar S. H., Rao K. V., Kurra N. (2025). Small.

[cit13] Gannett C. N., Kim J., Tirtariyadi D., Milner P. J., Abruña H. D. (2022). Chem. Sci..

[cit14] Banda H., Damien D., Nagarajan K., Raj A., Hariharan M., Shaijumon M. M. (2017). Adv. Energy Mater..

[cit15] Nishide H., Iwasa S., Pu Y.-J., Suga T., Nakahara K., Satoh M. (2004). Electrochim. Acta.

[cit16] Wang G., Dmitrieva E., Kohn B., Scheler U., Liu Y., Tkachova V., Yang L., Fu Y., Ma J., Zhang P., Wang F., Ge J., Feng X. (2022). Angew. Chem., Int. Ed..

[cit17] Peng Z., Yi X., Liu Z., Shang J., Wang D. (2016). ACS Appl. Mater. Interfaces.

[cit18] Luo C. (2023). Chem. Commun..

[cit19] Lee S. K., Zu Y., Herrmann A., Geerts Y., Müllen K., Bard A. J. (1999). J. Am. Chem. Soc..

[cit20] Lu Y., Cai Y., Zhang Q., Chen J. (2022). Adv. Mater..

[cit21] Hsiao S.-H., Liao W.-K., Liou G.-S. (2018). Polym. Chem..

[cit22] Yeh S. J., Tsai C. Y., Huang C.-Y., Liou G.-S., Cheng S.-H. (2003). Electrochem. Commun..

[cit23] Kim E. Y., Ta K. V. K., Chen F., Yang Z. Z., Yu Y., Luo C. (2024). Batteries Supercaps.

[cit24] Hsiao S.-H., Chen Y.-Z. (2017). J. Electroanal. Chem..

[cit25] Hu J., Liu X., Wang K., Wu M., Huang H., Wu D., Xia J. (2020). J. Mater. Chem. C.

[cit26] Li W., Dahn J. R., Wainwright D. S. (1994). Science.

[cit27] Zeng X., Hao J., Wang Z., Mao J., Guo Z. (2019). Energy Storage Mater..

[cit28] Jiang B., Huang T., Yang P., Xi X., Su Y., Liu R., Wu D. (2021). J. Colloid Interface Sci..

[cit29] Brown J., Karlsmo M., Bendadesse E., Johansson P., Grimaud A. (2024). Energy Storage Mater..

[cit30] Mayer J. M. (2023). J. Am. Chem. Soc..

[cit31] Agarwal R. G., Coste S. C., Groff B. D., Heuer A. M., Noh H., Parada G. A., Wise C. F., Nichols E. M., Warren J. J., Mayer J. M. (2022). Chem. Rev..

[cit32] Peper J. L., Mayer J. M. (2019). ACS Energy Lett..

[cit33] Bentley C. L. (2022). Electrochem. Sci. Adv..

[cit34] Ventosa E. (2021). Curr. Opin. Electrochem..

[cit35] Takahashi Y., Kumatani A., Munakata H., Inomata H., Ito K., Ino K., Shiku H., Unwin P. R., Korchev Y. E., Kanamura K., Matsue T. (2014). Nat. Commun..

[cit36] Yamamoto T., Ando T., Kawabe Y., Fukuma T., Enomoto H., Nishijima Y., Matsui Y., Kanamura K., Takahashi Y. (2021). Anal. Chem..

[cit37] Inomata H., Takahashi Y., Takamatsu D., Kumatani A., Ida H., Shiku H., Matsue T. (2019). Chem. Commun..

[cit38] Tao B., Yule L. C., Daviddi E., Bentley C. L., Unwin P. R. (2019). Angew. Chem., Int. Ed..

[cit39] Watkins T. S., Sarbapalli D., Counihan M. J., Danis A. S., Zhang J., Zhang L., Zavadil K. R., Rodríguez-López J. (2020). J. Mater. Chem. A.

[cit40] Gossage Z. T., Hui J., Zeng Y., Flores-Zuleta H., Rodríguez-López J. (2019). Chem. Sci..

[cit41] Hadjiivanov K. I., Panayotov D. A., Mihaylov M. Y., Ivanova E. Z., Chakarova K. K., Andonova S. M., Drenchev N. L. (2021). Chem. Rev..

[cit42] Adanigbo P., Malloy A. C., Laiq M., Wang L., Freschlin A. G., Vora P. M., Yu Y. (2026). J. Phys. Chem. Lett..

[cit43] Geng Y., Li H.-B., Wu S.-X., Su Z.-M. (2012). J. Mater. Chem..

[cit44] Janic I., Kakas M. (1984). J. Mol. Struct..

[cit45] Laviron E. (1979). J. Electroanal. Chem. Interfacial Electrochem..

[cit46] Zhang W., Rosendahl S. M., Burgess I. J. (2010). J. Phys. Chem. C.

[cit47] Lewis N. B., Bisbey R. P., Westendorff K. S., Soudackov A. V., Surendranath Y. (2024). Nat. Chem..

[cit48] Wang X., Tang W., Loh K. P. (2021). ACS Appl. Energy Mater..

[cit49] Pike S. J., Hutchinson J. J., Hunter C. A. (2017). J. Am. Chem. Soc..

[cit50] Tang M., Zhu S., Liu Z., Jiang C., Wu Y., Li H., Wang B., Wang E., Ma J., Wang C. (2018). Chem.

[cit51] Lin L., Lin Z., Zhu J., Wang K., Wu W., Qiu T., Sun X. (2023). Energy Environ. Sci..

[cit52] Wang C., Xu Y., Fang Y., Zhou M., Liang L., Singh S., Zhao H., Schober A., Lei Y. (2015). J. Am. Chem. Soc..

[cit53] Scholz F., Komorsky-Lovrić Š., Lovrić M. (2000). Electrochem. Commun..

[cit54] Deng H., Huang X., Wang L. (2010). Langmuir.

[cit55] Deng H., Dick J. E., Kummer S., Kragl U., Strauss S. H., Bard A. J. (2016). Anal. Chem..

[cit56] Liu C., Peljo P., Huang X., Cheng W., Wang L., Deng H. (2017). Anal. Chem..

[cit57] Barth B. A., Imel A. E., Zawodzinski T. (2025). J. Electrochem. Soc..

