# Supplementary material for: Resolving ion-coupled electron transfer in bipolar organic electrodes in aqueous electrolytes by single-particle electrochemistry
Source: Chem Sci. 2026 Sep 25. Online ahead of print. doi: 10.1039/d6sc04619c (PMC13614483; doi:10.1039/d6sc04619c)
Supplement: Supplementary file 1 [file SC-OLF-D6SC04619C-s001.pdf]

Supplementary Information (SI) for Chemical Science.
This journal is © The Royal Society of Chemistry 2026
Supplementary Information (SI) for Chemical Science.
This journal is © The Royal Society of Chemistry

Supplementary Information

Resolving Ion-Coupled Electron Transfer in Bipolar Organic Electrodes in Aqueous Electrolytes by Single-Particle Electrochemistry

George A. Akpowu[1], John R. Castilon[1], Pelumi Adanigbo[1], Andrea B. Brothers[1], Thida Samay[1], Victor Wang[1], Chao Luo[2], Yun Yu*[1]

[1] Department of Chemistry and Biochemistry, George Mason University, Fairfax, Virginia 22030, United States

[2] Department of Chemical, Environmental and Materials Engineering, University of Miami, Coral Gables, Florida 33146, United States

*Corresponding Author

E-mail: yyu26@gmu.edu

1. Experimental Methods

1.1 Chemicals and Materials

Perylenetetracarboxylic dianhydride (98 %) and Naphthalenetetracarboxylic dianhydride (>97%) were purchased from TCI. N1, N1-diphenylbenzene-1,4-diamine (99.92%), imidazole (99%), ethyl alcohol (99.5%), and N, N-Dimethylformamide (DMF) (99.8%) were purchased from Sigma-Aldrich. Tetrahydrofuran (99.9%) was purchased from Spectrum Chemical. N-methyl-2-pyrrolidone (NMP), poly(vinylidene fluoride), carbon black (Super P, >99%), sodium dihydrogen phosphate, disodium phosphate, sodium perchlorate, perchlorate acid, sulfuric acid, and sodium hydroxide were received from Thermo Fisher Scientific. Graphite and highly oriented pyrolytic graphite (HOPG) were received from HQ Graphene and SPI Supplies, respectively.

1.2 OEM Synthesis

In the PDI-TPA synthesis, perylenetetracarboxylic dianhydride (1 mmol) was dissolved in the liquid imidazole under inert gas, N_2, at ~90°C. N1, N1-diphenylbenzene-1,4-diamine (4 mmol) was transferred into a round bottom flask when the temperature reached ~150°C. The reaction temperature was then maintained at ~170 °C for 24 h. After completion, the reaction mixture was gradually cooled to ~60 °C to allow crystallization. The resulting solid was collected and washed with ethanol. The material was filtered under vacuum and further purified by sonication in DMF (10 mL) for ~10 min, repeated four times, followed by sonication in ethanol (10 mL) three times for ~10 min each. The product was then filtered and dried in a vacuum oven at 80 °C overnight.

The NDI-TPA synthesis followed a similar procedure. Naphthalenetetracarboxylic dianhydride (1 mmol) was dissolved in liquid imidazole and 10 mL DMF then stirred for 24 h with magnesium sulfate for dehydration. To provide an inert atmosphere, N_2 gas was pumped into the reaction vessel for one hour. Subsequently, the solution was heated until it reaches ~140°C. The N1, N1-diphenylbenzene-1,4-diamine was dissolved in 5 mL of DMF solvent, was subsequently injected into the naphthalenetetracarboxylic dianhydride solution. The temperature was then maintained at ~140°C for 24 hours. After the temperature was reduced to room temperature, the precipitate was washed and filtered using tetrahydro furan, DMF, and ethanol. The obtained material was dried at 80°C in the vacuum oven overnight.

The synthesized PDI-TPA powders and graphite (mass ratio 7:1) were ground for 30 min, then mixed with carbon black and polyvinylidene fluoride (7:1:1) for another 30 min. These additives provide mechanical cohesion and improve electronic connectivity between PDI-TPA

particles and the current collector, but do not contribute redox current in the relevant potential window. The mixture was dispersed in NMP, applied to HOPG, and dried at 80 °C for ~2 h. This same method was repeated for the preparation of the NDI-TPA on HOPG.

1.3 OEM Characterization

The Raman spectra were recorded with a Horiba XploRa Plus Raman microscope with a 532 nm laser source. The acquisition time was set to 2 seconds.

The room-temperature powder X-ray diffraction (XRD) patterns were recorded using a Rigaku MiniFlex-600 benchtop powder diffractometer with Cu Ka (λ = 1.5418 Å) radiation. The data was collected for 20 minutes by increasing the scattering angle 2θ from 5° to 60°.

The thickness and the topography of the OEMs were determined with atomic force microscopy (NX12, Park System) operated in non-contact mode.

1.4 SECCM Experiments

Single channel nanopipettes were fabricated from borosilicate glass capillaries with filament, with an outer diameter of 1.00 mm and an inner diameter of 0.58 mm (BF-100-58-15, Sutter Instruments). The capillaries were pulled using a laser puller (P-2000, Sutter Instruments) with the following parameters: Heat = 320, Filament = 0, Velocity = 10, Delay = 150, and Pull = 150. The geometries of the pulled nanopipettes were characterized in Figure S9, which shows that the nanopipette orifice is approximately 300–400 nm in diameter. The nanopipette was then filled with electrolyte solutions of desired pH. An Ag/AgCl wire was inserted into the channel of the nanopipette, which serves as the quasi-reference counter electrodes (QRCE). The potential-stability test in Figure S10 showed essentially identical redox potentials over the five-hour experimental duration, confirming sufficient QRCE stability under the SECCM conditions used in this work. The potential of our Ag/AgCl QRCE is 233±17 mV vs. commercial Ag/AgCl (1 M KCl) reference electrode in phosphate buffer solutions, as determined by our potentiometry calibration.

The SECCM experiments were performed using a NX12 SICM module (Park Systems). The nanopipette was positioned over the area of interest on the HOPG with the help of optical microscope. With a bias of 0.5 V applied, the probe gradually approaches the sample at a speed of 0.1 μm/s until a 3-pA threshold current was achieved due to the contact formed between the meniscus and the sample surface. Electrochemical mapping, conducted in a hopping mode, was employed to visualize the activity of individual OEM particles. In a hopping mode, the nanopipette forms a meniscus cell at one location, collects the electrochemical response, retracts, moves

laterally to the next position, and repeats the process. An initial SECCM map is first collected. Specific regions of interest are then selected by correlating the electrochemical map with the corresponding optical image, and these locations can be revisited for additional measurements, such as scan-rate-dependent voltammograms. The effective spatial resolution is determined by the spacing between adjacent measurement points, which was chosen to be larger than the meniscus contact size to avoid overlap between neighboring measurements.

The phosphate buffers were prepared using Henderson–Hasselbalch calculations with appropriate H_3PO_4, NaH_2PO_4, and Na_2HPO_4 ratios. The Na^+ concentration was adjusted to account for the H^+ concentration at each pH, bringing the total free cation concentration to 0.5 M. The buffer pH was adjusted using the corresponding phosphate salt or 0.5 M NaOH for basic solutions. The final pH of each buffer was verified with a calibrated pH meter before use.

Figure S11 shows the effects of the additives on the observed SECCM heterogeneity.

2. Supplementary figures

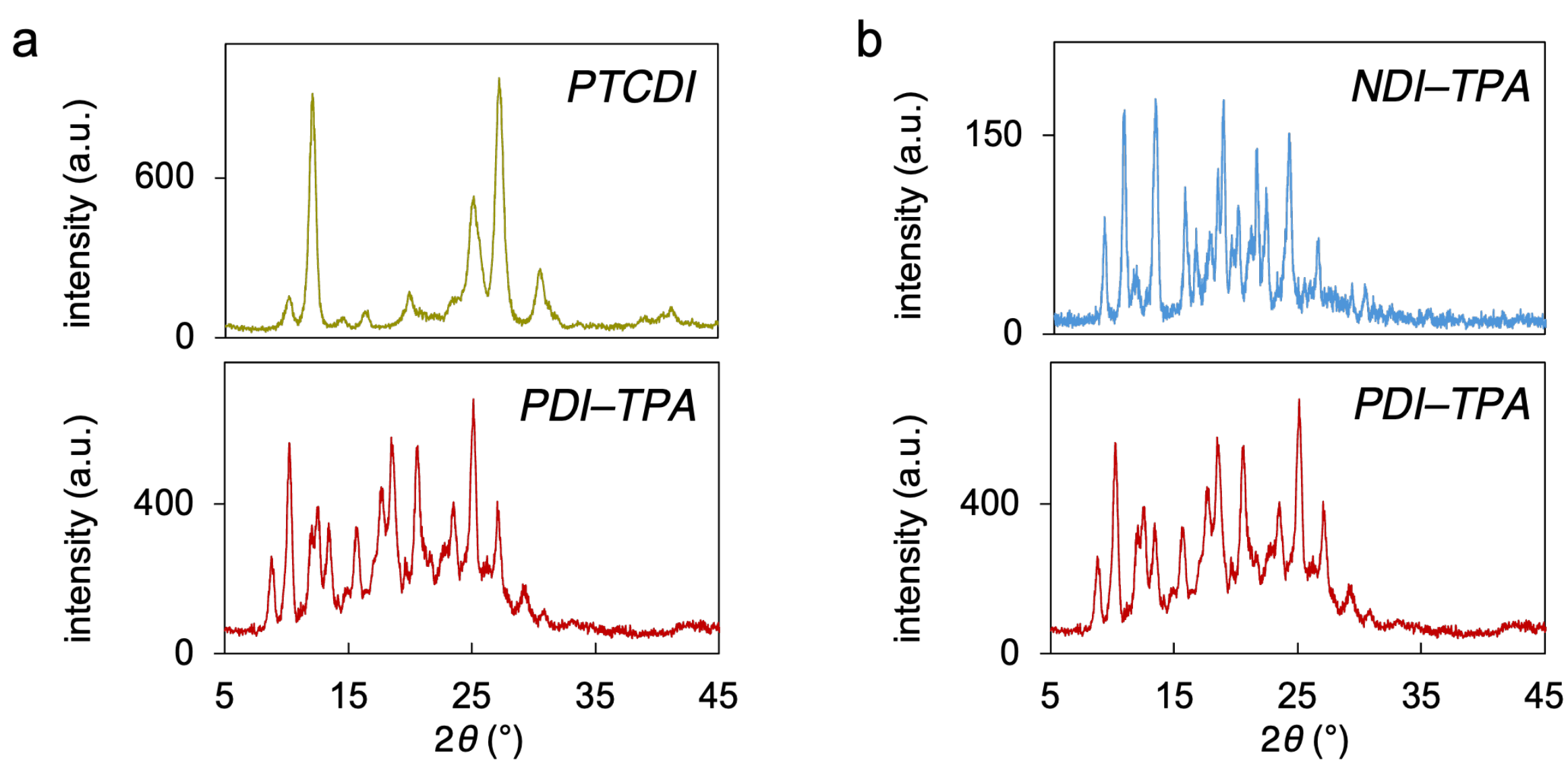

Figure S1. (a) X-ray diffractograms of PDI-TPA and 3,4,9,10-perylenetetracarboxylic diimide (PTCDI). Compared to PDI-TP, PTCDI consists of the same planar perylene diimide structure without the bulky TPA substituents. (b) X-ray diffractograms of PDI-TPA and NDI-TPA.

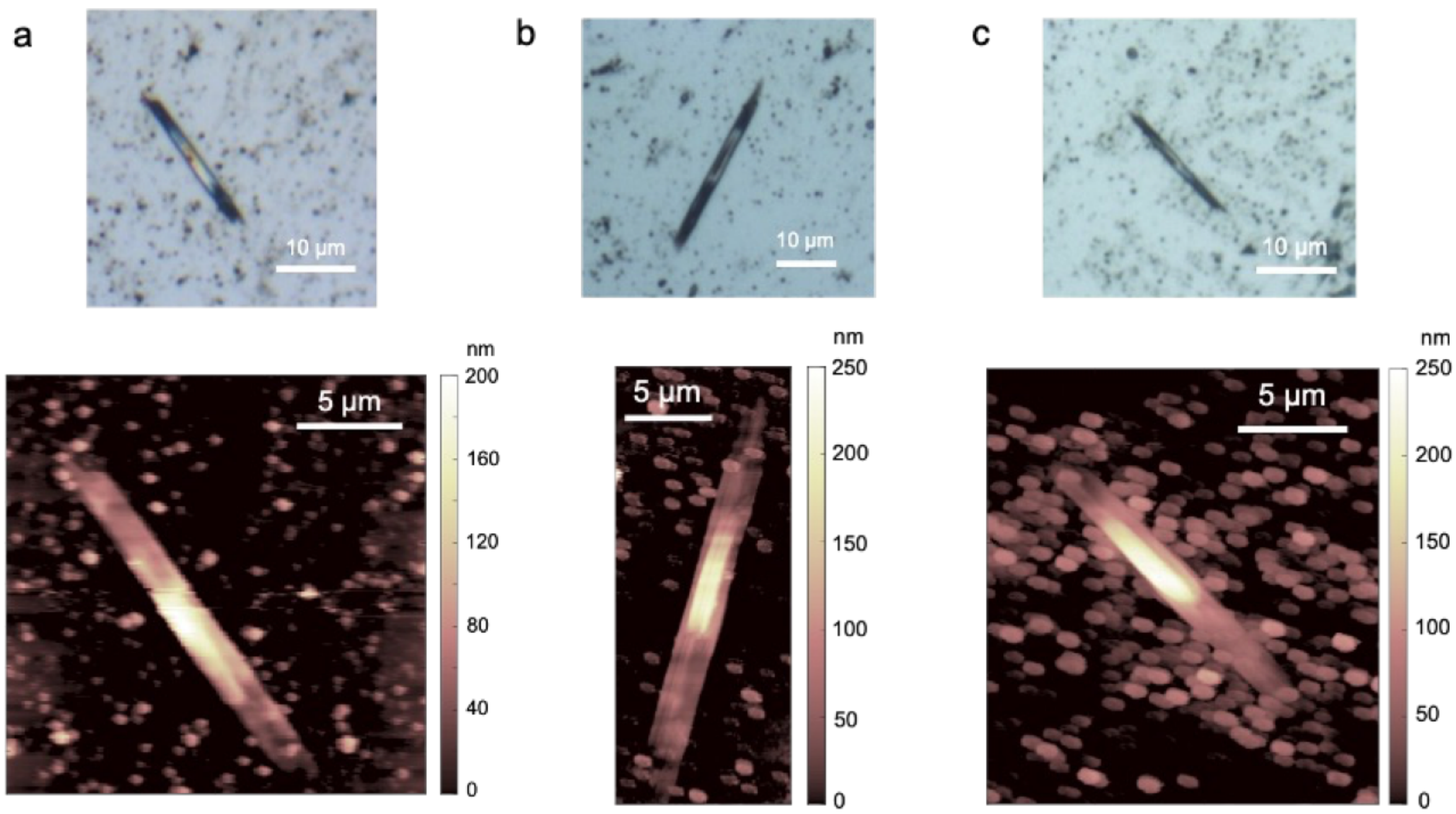

Figure S2. (a-c) Optical micrographs (top) and non-contact AFM topographic images (bottom) of three PDI-TPA particles deposited on HOPG surface.

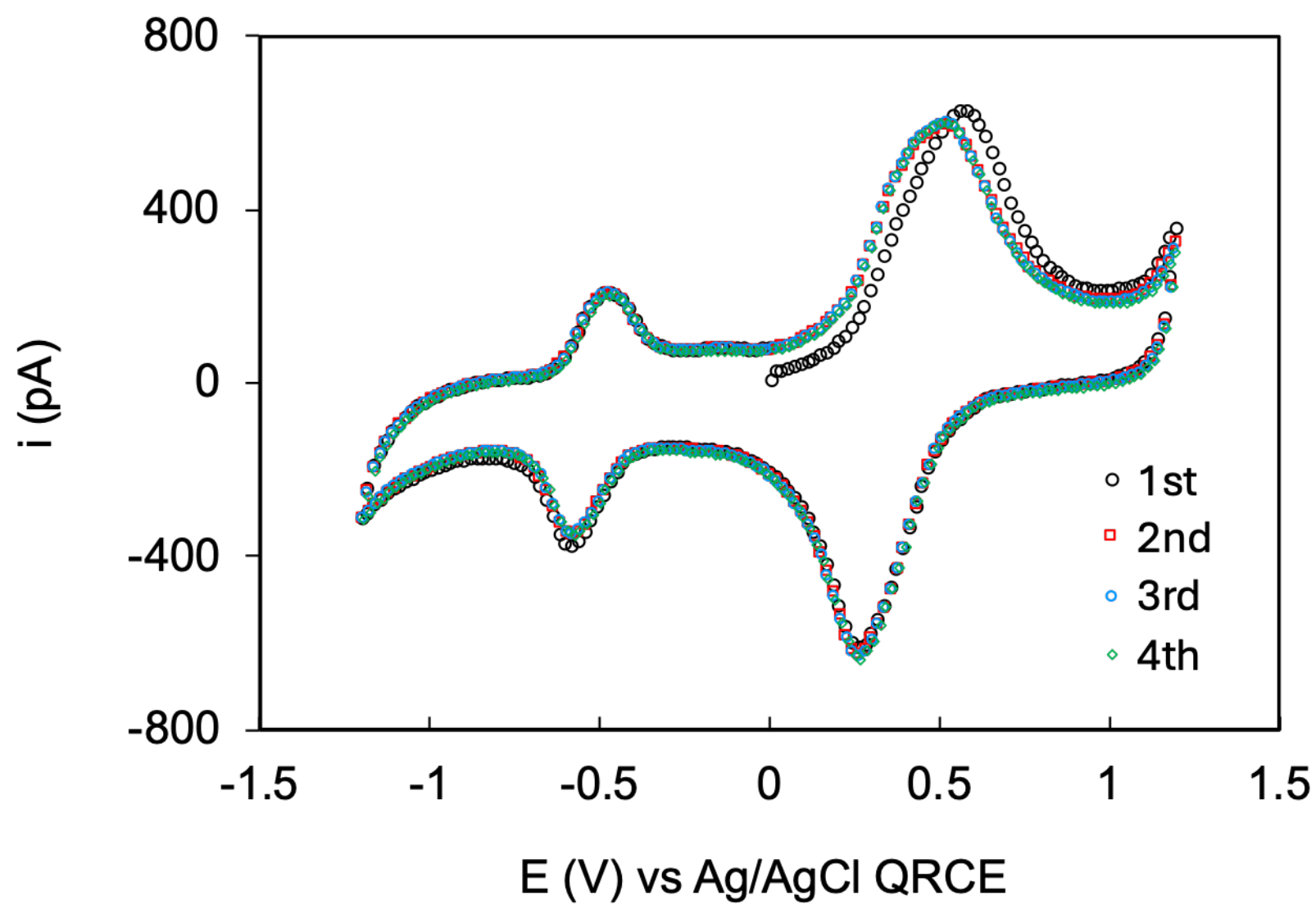

Figure S3. Four consecutive local voltametric scans recorded at PDI-TPA particles in phosphate buffers containing 0.50 M Na^+ at pH 1.6 with a scan rate of 1 V/s. The traces for cycles 1–4 are shown as black (1st), red (2nd), blue (3rd), and green (4th) respectively.

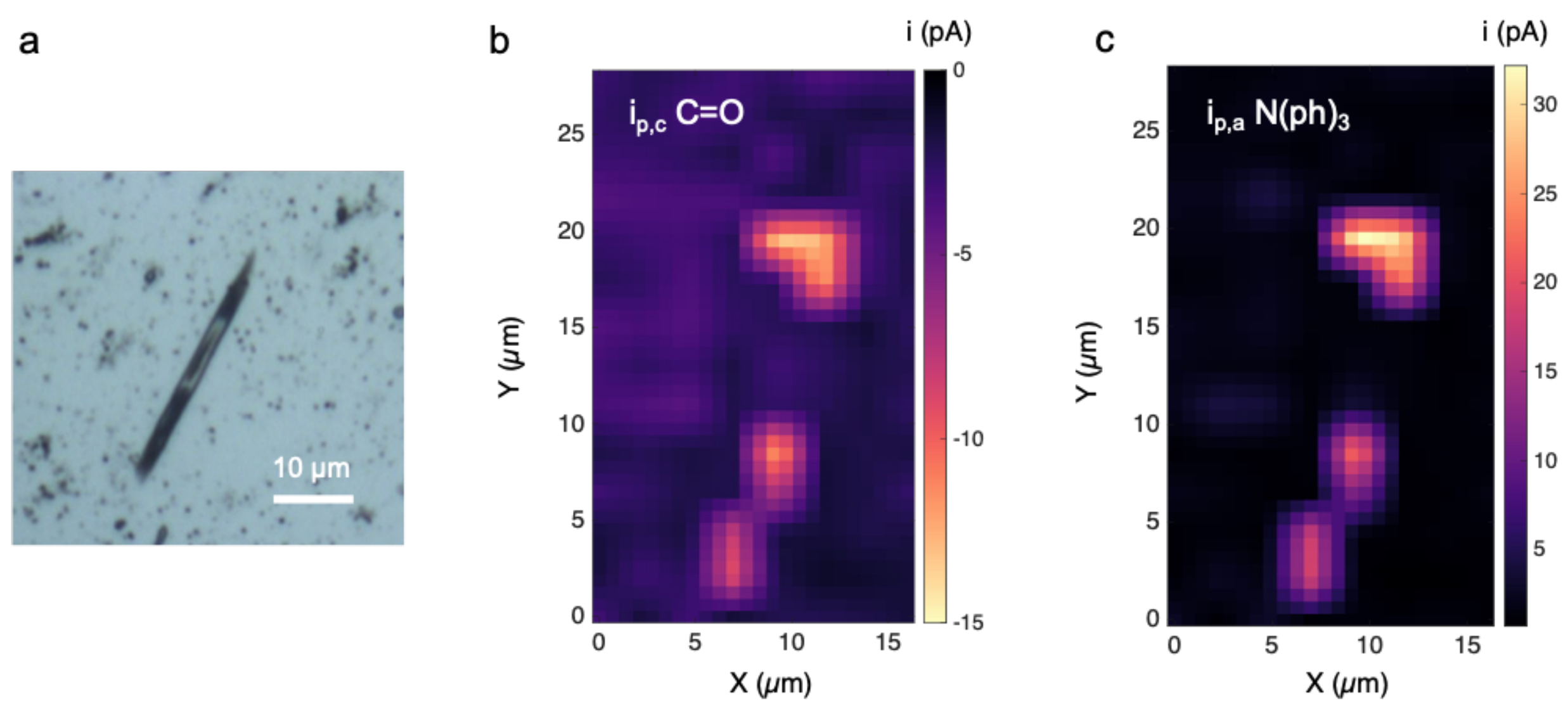

Figure S4. (a) Optical image of a PDI-TPA particle. (b-c) SECCM current maps of the PDI-TPA particle collected at the carbonyl cathodic peak (b) and the amine anodic peak (c). The aqueous electrolyte contains pH 1.6 phosphate buffer and 0.45 M Na^+.

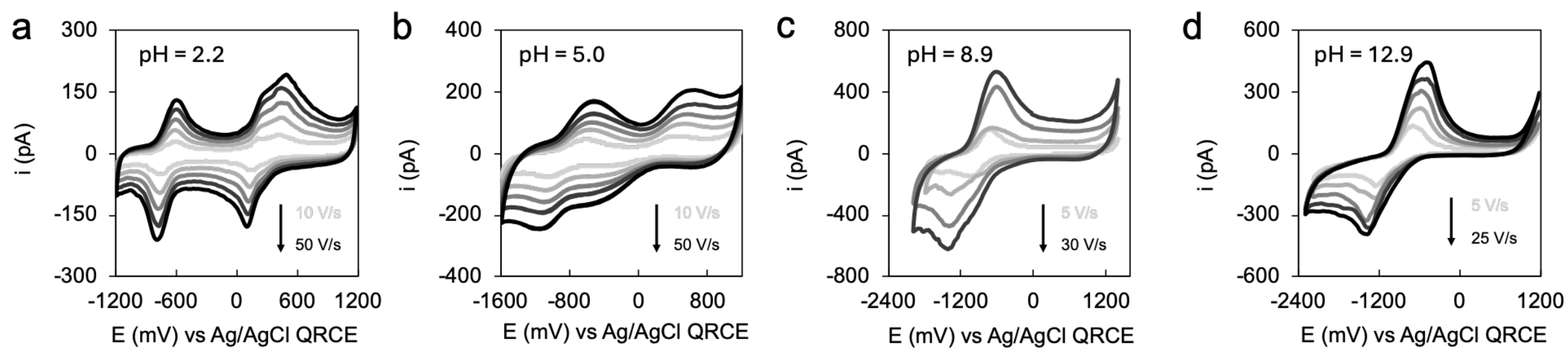

Figure S5. (a-d) Representative local voltammograms recorded at PDI-TPA with various scan rates in phosphate buffers containing 0.50 M Na^+ at pH 2.2 (a), 5.0 (b), 8.9 (c), and 12.9 (d).

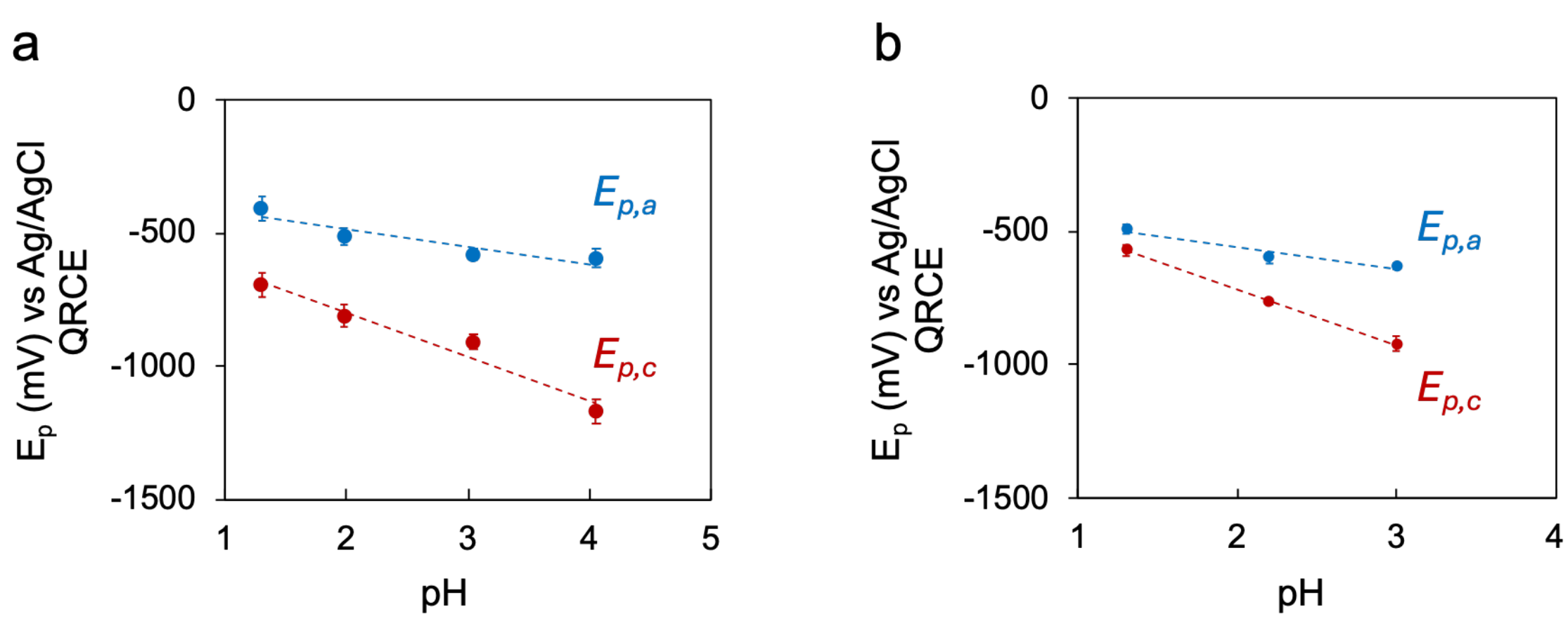

Figure S6. (a-b) Plot of the cathodic peak potential ($E_{p,c}$) and anodic peak potential ($E_{p,a}$) of PDI carbonyl redox as a function of the acidic pH with scan rate of 10 V/s (a) and 0.5 V/s (b). In both time scales, $E_{p,c}$ and $E_{p,a}$ show asymmetric dependence on pH.

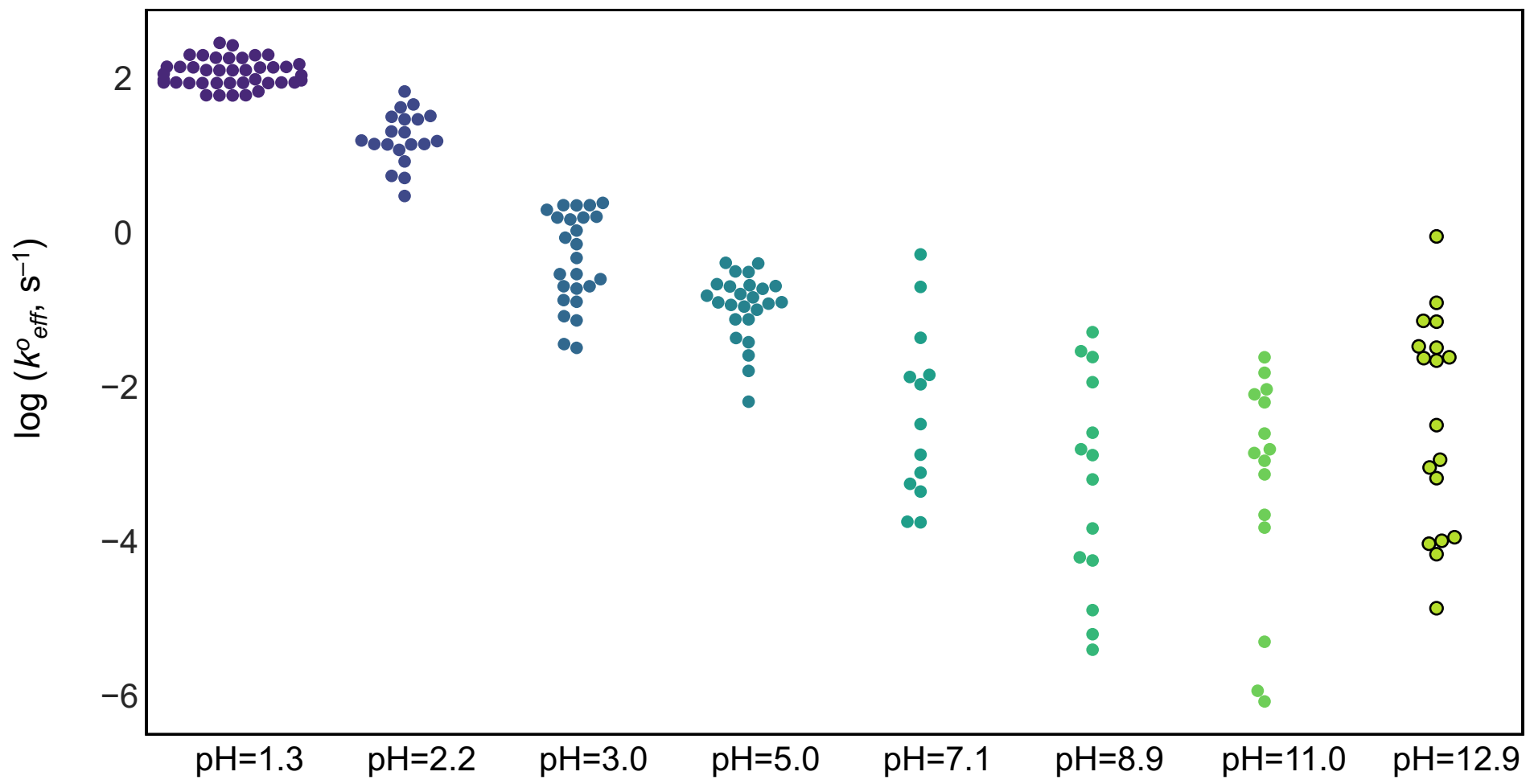

Figure S7. Swarm plot showing the distribution of log(k_{eff}) for PDI carbonyl redox as a function of pH. Each data point represents an individual measurement, with the horizontal spread illustrating data density at each condition.

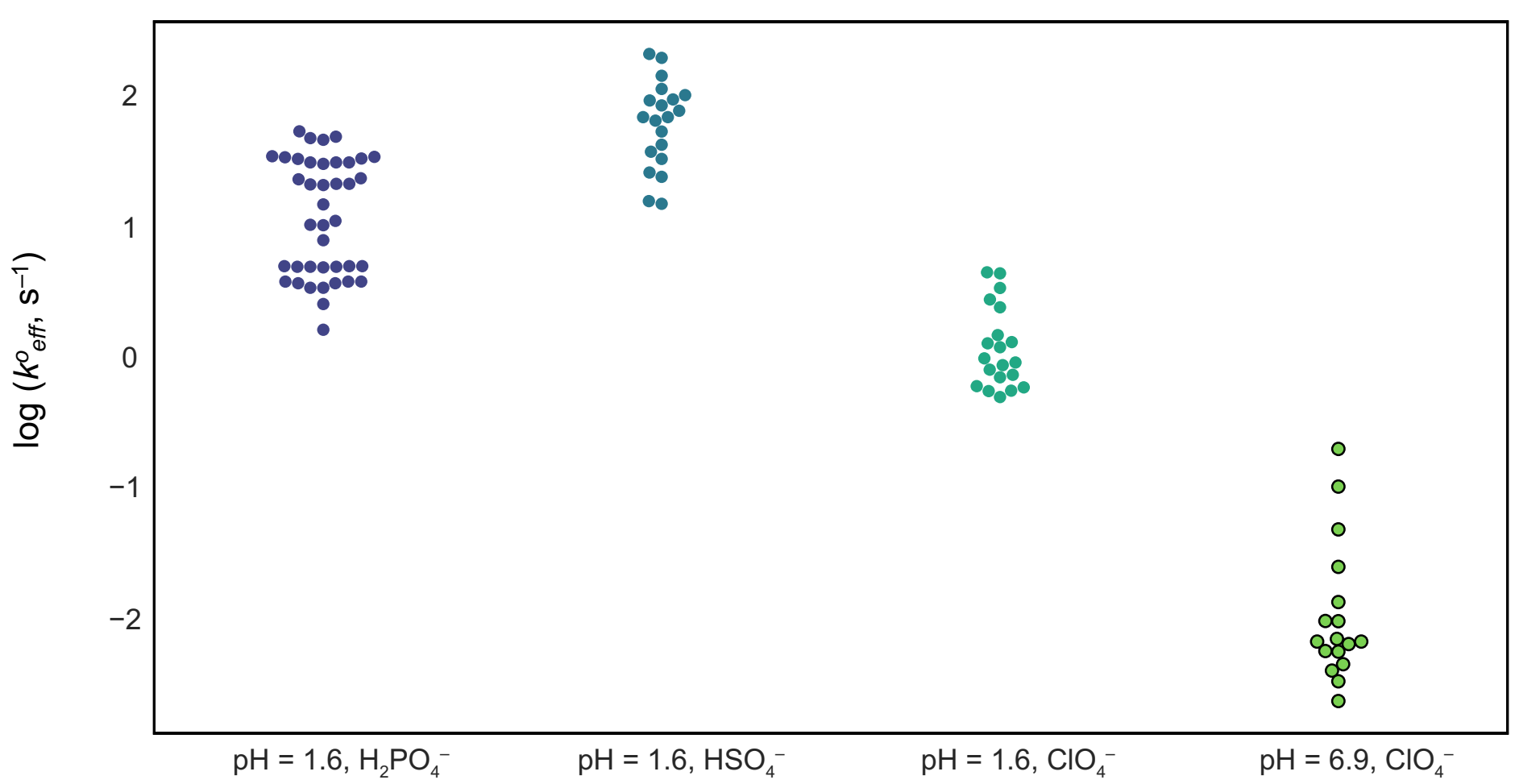

Figure S8. Swarm plot showing the distribution of log(k_{eff}) for TPA redox. Each data point represents an individual measurement, with the horizontal spread illustrating data density at each condition.

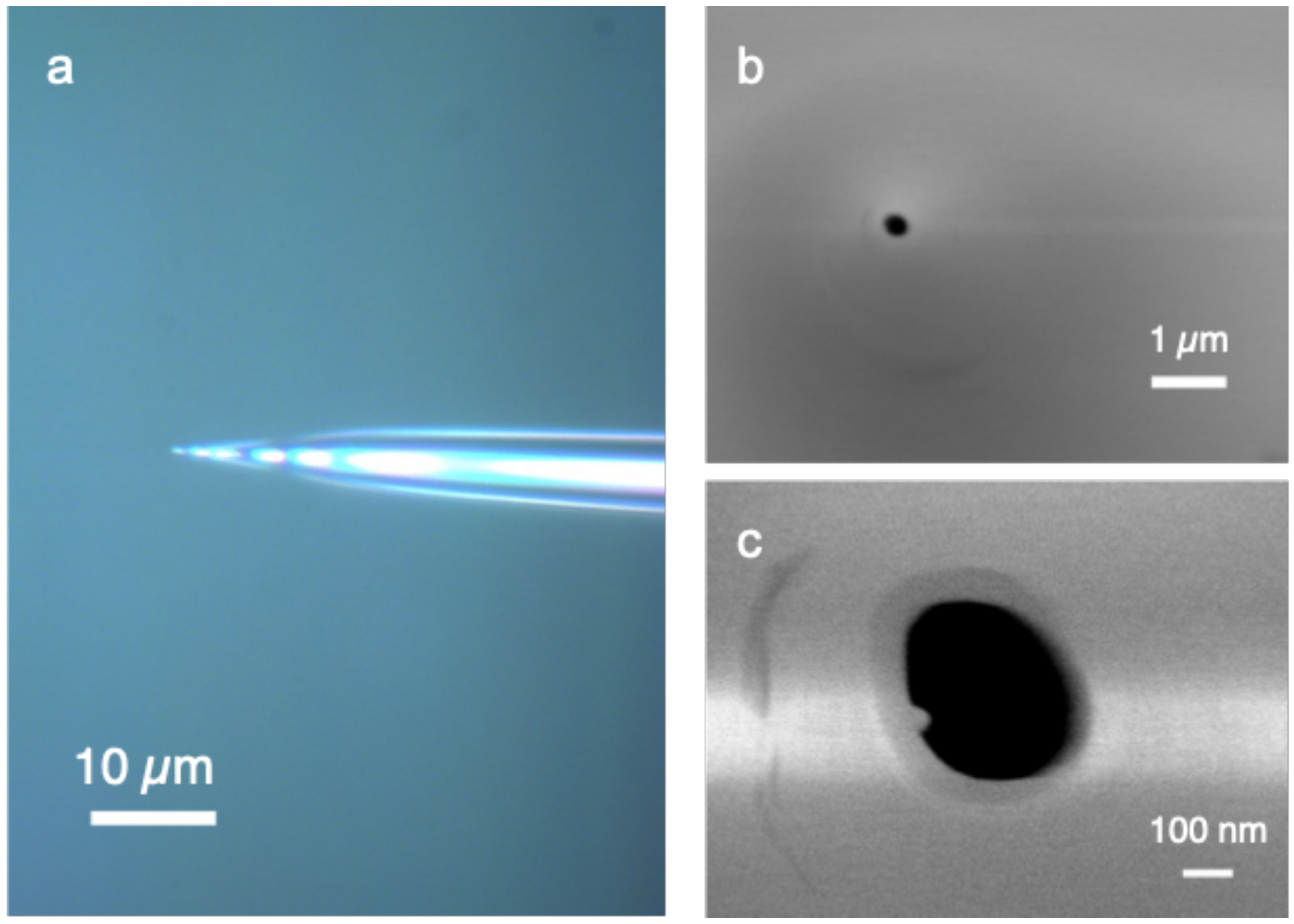

Figure S9. (a) Optical micrograph of a representative nanopipette, showing a tapered length of approximately 20 μm and an estimated cone angle of 7.5°. (b,c) SEM images of the nanopipette orifice shown in panel (a). The outer and inner diameters of the orifice are approximately 417 nm and 348 nm, respectively.

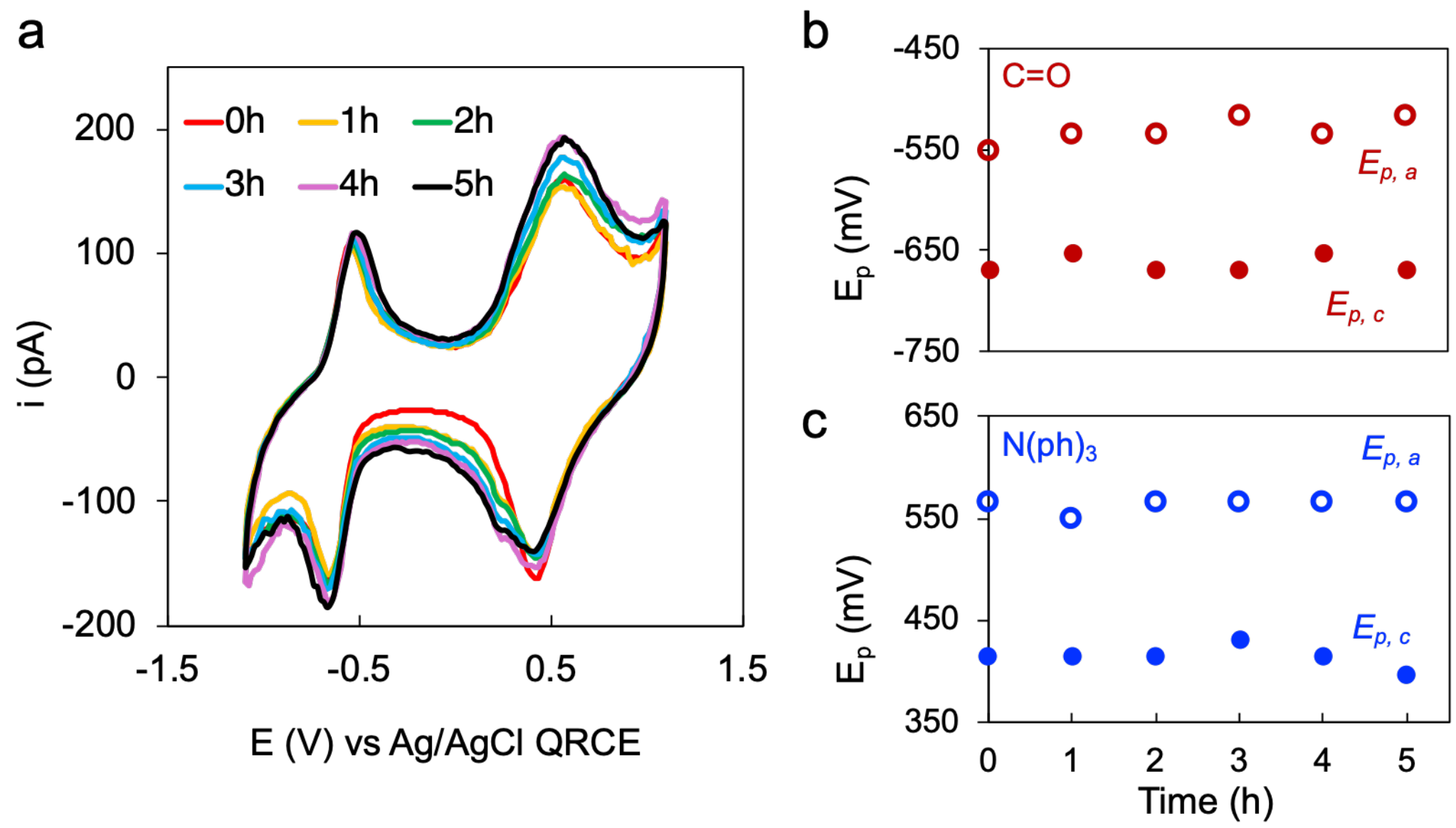

Figure S10. (a) Local voltammograms collected at the same PDI-TPA location every hour over a 5 h period in pH 1.6 phosphate buffer. (b,c) Time-dependent $E_{p,a}$ and $E_{p,c}$ for the C=O and $N(Ph)_3$ redox processes, respectively. The variations in peak potentials over the 5 h measurement are comparable to the error bars shown in Figure 4a, confirming sufficient potential stability.

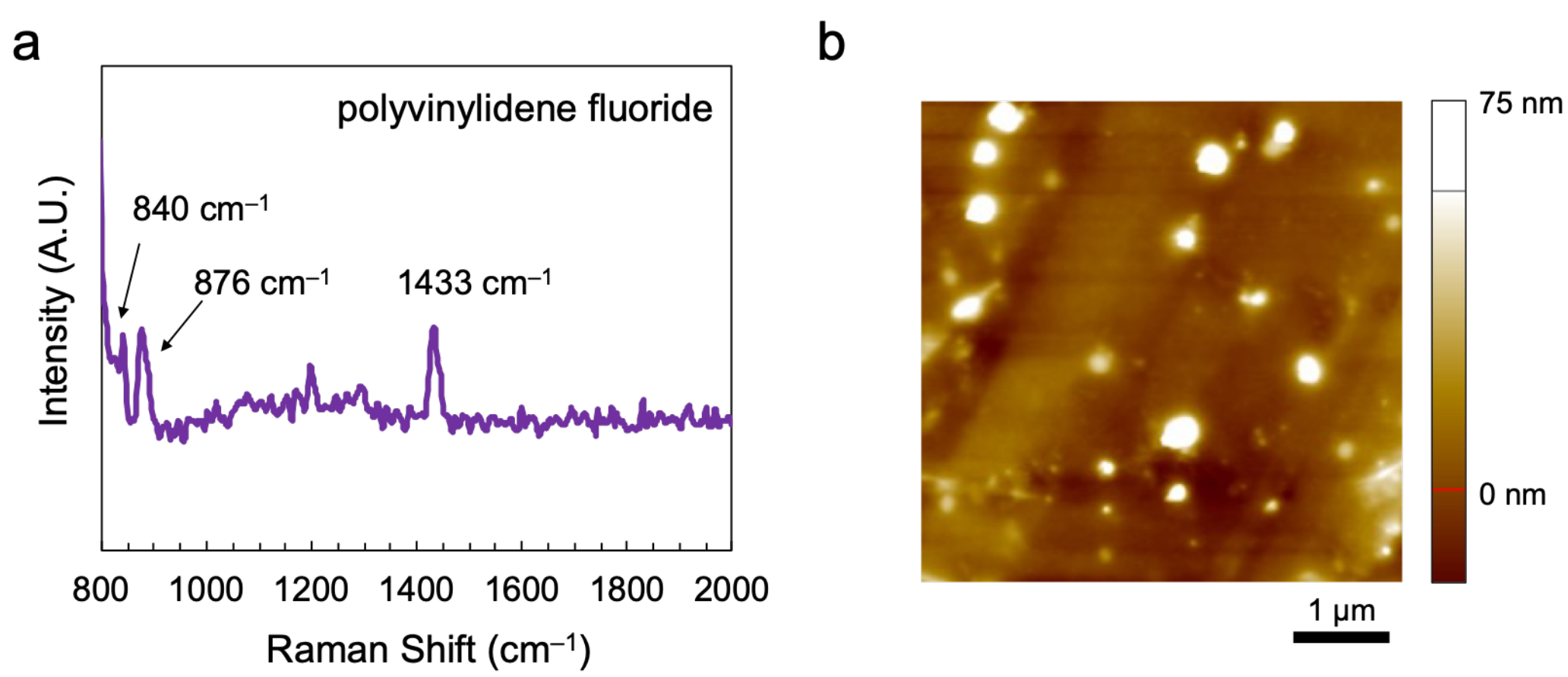

Figure S11. (a) Raman spectrum of the polyvinylidene fluoride reference, showing features at approximately 840, 876, and 1433 cm^{-1} that are not detectable in the spectra of the PDI-TPA particles in Figure 1e. (b) Non-contact AFM topographic images of the polyvinylidene fluoride-graphite-carbon black mixture deposited on HOPG surface (60–360 nm in size). The additives are expected to have minimal impact on the observed SECCM heterogeneity.

3. Determination of the effective charge transfer coefficient

The analytical framework developed by Lewis *et al* [1] is shown in Equation S1:

$$k_{app}(pH) = k_a^0 10^{-(1-\alpha_a)pH} + k_b^0 10^{\alpha_b(pH-pK_w)} \qquad \text{S1}$$

where $k^0{}_a$ and $k^0{}_b$ are the standard rate constant for PCET reactions with hydronium and water as the principal proton donors, respectively. α_a and α_b are charge transfer coefficient for the respective reactions. pK_w is the dissociation constant of water. In our OEM system, the second term is omitted. Equation S1 is rewritten as Equation S2:

$$k_{eff} = k_{app}(pH) = k^0 10^{-(1-\alpha)pH} \qquad \text{S2}$$

Equation S2 can be rearranged to Equation S3:

$$\log(k_{eff}) = logk^0 + (\alpha - 1)pH \qquad \text{S3}$$

The slope of $\log(k_{eff})$ vs pH dependence is (α–1). Figure 4d shows a slope of α–1= −0.73, yielding α = 0.27. The *y* intercept is $\log k^0 = 2.7$, yielding $k^0 = 502\ s^{-1}$.

4. Reference

(1) Lewis, N. B.; Bisbey, R. P.; Westendorff, K. S.; Soudackov, A. V.; Surendranath, Y. A Molecular-Level Mechanistic Framework for Interfacial Proton-Coupled Electron Transfer Kinetics. *Nat. Chem.* 2024, 16, 343-352
